# Comparative nutritional and antioxidant profiling of Assam honeys: unveiling the untapped bioactivity of stingless bee honey

**DOI:** 10.3389/fnut.2025.1737497

**Published:** 2025-12-16

**Authors:** Partha Pratim Gyanudoy Das, Mukul Kumar Deka, Abhibandana Das, Rituraj Borah, Mousumi Bharali, Shimantini Borkataki, Pradeep Kumar, R. Karthik, Titikshya Kashyap, Kereyagalahalli Mallaiah Kumaranag, Sachin Suresh Suroshe

**Affiliations:** 1 AICRP on Honey Bees and Pollinators, Department of Entomology, Assam Agricultural University, Jorhat, India; 2 Department of Forestry, North Eastern Regional Institute of Science and Technology (NERIST), Nirjuli, India; 3 Department of Entomology, Assam Agricultural University, Jorhat, India; 4 Department of Botany, University of Lucknow, Lucknow, India; 5 College of Life Science and Biotechnology, Korea University, Seoul, Republic of Korea; 6 Central Potato Research Institute (Regional Station), Jalandhar, India; 7 Project Coordinating Unit, AICRP on Honey Bees and Pollinators, Division of Entomology, ICAR-Indian Agricultural Research Institute, New Delhi, India

**Keywords:** stingless bee honey, physicochemical properties, DPPH, ABTS assay, antimicrobial efficacy, Northeast India

## Abstract

Honey is a nutritionally rich natural product with functional and bioactive relevance, yet species-specific comparative data from Northeast India remain limited. This study evaluated the physicochemical composition, mineral profiles, antioxidant, and antimicrobial potential of *Apis cerana*, *A. mellifera*, *A. dorsata*, and *Tetragonula iridipennis* honeys from Assam, with special emphasis on the bioactivity of stingless bee honey (*T. iridipennis*). Significant interspecific variation was observed: *T. iridipennis* honey exhibited elevated diastase activity (19.63 DN), proline (1,286 mg/kg), and mineral richness, along with the highest total phenolic (84.24 mg GAE/100 g) and flavonoid (21.20 mg QE/100 g) content. These biochemical traits corresponded to superior antioxidant capacity, with the lowest EC₅₀ values in DPPH (51.55 μL/mL) and ABTS (47.23 μL/mL) assays, and the broadest antibacterial spectrum, inhibiting *Salmonella Typhi*, *Shigella flexneri*, *Streptococcus pyogenes*, and *S. mutans*. *In-vitro* cytotoxicity assays further demonstrated notable inhibitory effects of *T. iridipennis* honey on cell viability, consistent with its high antioxidant potential. This study provides the first comprehensive biochemical baseline for Assam honeys and highlights stingless bee honey as a promising candidate for sustainable functional food development and bioactive compound exploration.

## 1 Introduction

Honey is one of nature’s most ancient and nutritionally rich foods, valued across civilizations for its flavor, energy, and health-promoting attributes. Beyond its role as a natural sweetener, honey represents a complex biochemical matrix of sugars, enzymes, minerals, and bioactive compounds that contribute to human nutrition and wellness. Historical records indicate that honey has been used in Egyptian, Greek, Ayurvedic, and Traditional Chinese medicine since time immemorial (1–3). In particular, honey from stingless bees has been widely valued as an ethnomedicine by diverse communities worldwide (4, 5) due to its considerable content of several bioactive compounds. In addition to carbohydrates (60–85%) and water (12–23%), honey also contains small amounts of other compounds, such as organic acids, minerals, vitamins, enzymes, proteins, amino acids, volatile compounds and bioactive substances like phenols and flavonoids (6, 7). The richness in nutritional bioactive compounds is what makes honey an effective treatment against a number of diseases such as wounds, skin ulcers, eczema, urinary, respiratory and gastrointestinal disease etc. (8). Further, many studies have proved that honey possess antioxidant, antimicrobial, antiviral, antiparasitory, anti-inflammatory, antimutagenic and anticancer effects (9). Honey’s ability to inhibit clinically significant microorganisms has been extensively documented in numerous studies (10–14). Honey is said to include a variety of polyphenols having antioxidant properties. Certain polyphenols present in honey, including apigenin, galangin, quercetin, acetin, kaempferol, pinocembrin, pinobanksin, and phenolic acid phenyl ester, have shown promise as potential candidates in the field of pharmacology (15). This affirms that honey rich in antioxidants can be considered as a valuable functional food with potential health-promoting properties. Thus, honey is not merely a natural sweetener, but a complex bioactive matrix whose nutritional and therapeutic potential continues to inspire both traditional use and modern scientific exploration.

In the context of beekeeping, North-East India is considered as one of the potential regions under Indo-Burma biodiversity hotspot due to the availability of rich bee flora in this area. The climatic condition of this region suits floral proliferation which is also signified by the extensive greenery and forest land of this region. Out of numerous flowering plants of this region, 2,526 species are endemic to this locality (16). The region is well known as ‘cradle of flowering plants’ and many economically important medicinal and aromatic plants are found in different parts of the region. Hence, availability of enormous numbers of flowering plants is the key factor to bloom beekeeping sector in this locality, especially in the state like Assam. The beekeepers of Assam mainly focus on commercial and scientific rearing of Asian honey bee, *Apis cerana* as well as European honey bee, *Apis mellifera* in different parts of the state. Parallelly, the rock bee, *Apis dorsata* is also a commonly found wild bee species and hunting of such bees is very common in the rural areas of Assam for greater honey and wax production potentials of this species. Apart from the three *Apis* species (stinging honey bees), *Tetragonula iridipennis*- a stingless bee species belonging to the tribe Meliponini is also prevalent in this region and known for its high antioxidant content and ethnomedicinal values. Harvesting of stingless bee honey from the forestland is also a common practice among tribal communities of Northeast India, where it is traditionally valued as an ethnomedicine for ailments such as burns, internal wounds, eye infections, diarrhea, ulcers etc. and is also consumed for its perceived ability to provide sustained energy, underscoring its immense therapeutic and nutraceutical potential (17, 18). The nutritional composition of honey will differ based on the geographical and botanical variation of flora and bee species (19). Given the distinct foraging preferences and physiological traits of different bee species, their honeys are expected to differ in composition and bioactivity. Despite growing global interest in honey bioactivity, comparative evaluations of honeys from different bee species in North-East India remain scarce, particularly with respect to stingless bee honey. Addressing this gap is important for establishing scientific baselines that can support its future application in nutraceuticals, functional foods, and evidence-based ethnomedicine. Hence, this study aims to systematically evaluate the physicochemical characteristics, mineral content, antioxidant, and antimicrobial properties of honeys from four key bee species- *A. cerana*, *A. mellifera*, *A. dorsata*, and *T. iridipennis*- collected from Jorhat, Assam. Special emphasis is placed on the stingless bee honey, which holds significant traditional and bioactive relevance, thereby establishing a scientific baseline for its potential application in sustainable functional food development.

## 2 Materials and methods

### 2.1 Extraction of honey

**Figure 1:**
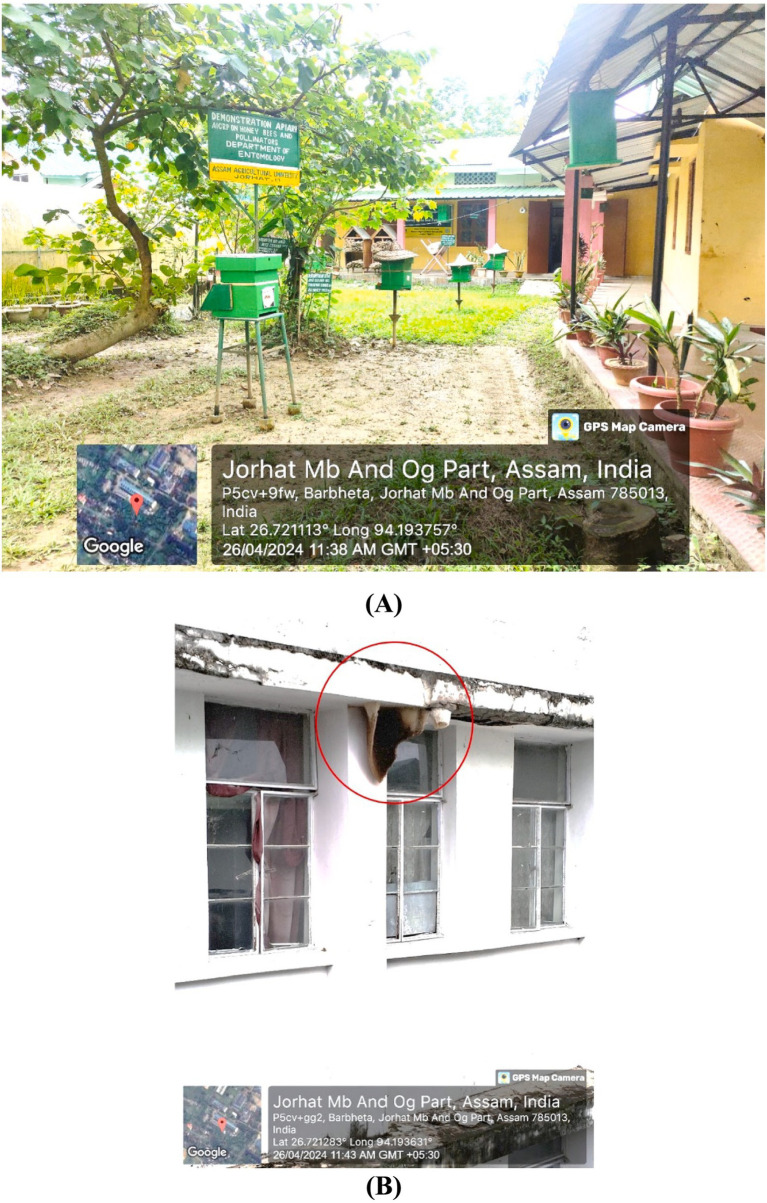
Bee colonies at Department of Entomology, AAU, Jorhat, Assam. **(A)** Demonstration apiary. **(B)** Rock bee colony adjacent to demonstration apiary.

Ripe and matured honey of *Apis cerana*, *Apis mellifera*, and *Tetragonula iridipennis* was extracted in April 2024 from the demonstration apiary of the Department of Entomology, Assam Agricultural University (AAU), Jorhat, Assam (26.721113° N, 94.193757° E), where domesticated hives of aforementioned species were maintained (Figure 1A). Honey of rock bee, *Apis dorsata* from the same period was collected from naturally constructed hives located adjacent to the apiary site (Figure 1B). Five hundred grams (500 g) of clean honey was sampled from each species and labeled properly for further analysis.

### 2.2 Evaluation of physiochemical and biochemical properties of honey samples

Specific gravity, moisture (%) and total reducing sugar (%) of honey samples were evaluated by the method IS 4941 (20). Sucrose (%), F/G ratio and pollen count (count/g) were determined according to the AOAC method (21). Total ash (%) was determined by the method IS 4941 (20). Free acidity (mEq Acid/1000 g), EC (mS/cm) and pH were evaluated following Harmonized methods of the International Honey Commission International Honey Commission (22). Further AOAC methods were followed to determine Hydroxy Methyl Furfural content (mg/kg) (23), Diastase activity (DN) (24) and Proline (mg/kg) (25). Further, physical color was also recorded with respect to each four honey samples.

### 2.3 Evaluation of elemental profile of honey sample

Elemental profile of four honey samples were determined by Flame Atomic Absorption Spectrometry (model name: AAS-iCE 3,000, Thermo Fisher Scientific, SOLAAR Software) following the method given by Barbeş et al. (26) with slight modification. Each honey sample (10 g) with three replicates were subjected to dry ashing following desiccation (105 °C for 12 h), slow thermal treatment (50 °C/h) followed by final calcination step (450 °C/10 °C). The resulting white ashes were treated with 1 mL concentrated nitric acid finally transferred to volumetric flasks (100 mL) and volume makeup was done with deionized water. The solution was then aspirated into the oxygen-acetylene flame of the instrument which was calibrated employing atomic absorption standard solutions (Inorganic ventures™, AACA1) of different metal salts. The final mineral concentration for each element of all the honey samples were expressed as mg/100 g of honey sample.

### 2.4 Evaluation of total phenol content (TPC) and total flavonoid content (TFC)

The total phenolic content (TPC) of four honey samples were determined by using Folin–Ciocalteu method described by Singleton et al. (27) with some modifications (28, 29). For TPC, 0.5 g of honey sample and 2.5 mL of Folin Ciocalteu reagent (2 N) were combined, and the mixture was incubated for 5 min. The mixture was again incubated for 2 hours at 25 °C after 2 mL of sodium carbonate (Na_2_CO_3_) solution (75 g/L) was added. A UV–Visible spectrophotometer (Systronics Double Beam Spectrophotometer: 2203) was then used to measure the solution’s absorbance at 760 nm. By comparing the absorbance values of the honey samples with the calibration curve derived from gallic acid (GA) standards solutions (*R^2^* = 0.9948) ranging from 0 to 100 mg/L, the TPC was determined and mean of three replications was expressed in milligrams of gallic acid equivalent (GAE) per 100 g of honey sample (mg GAE/100 g).

TFC was determined with the help of standard aluminum chloride method (30). Briefly, each honey sample (0.01 mg/mL) was combined with 5 mL of 2% aluminum chloride (AlCl_3_) in methanol solution and incubated for 10 min at 25 °C. A UV–Visible spectrophotometer (Systronics Double Beam Spectrophotometer: 2203) was used to measure the flavonoid-aluminum complex at 415 nm. Honey solution (5 mL) mixed with 5 mL methanol without AlCl3 was taken as blank. The absorbance values were then compared to a calibration curve that was created using quercetin standards (*R^2^* = 0.9867) that ranged in concentration from 0 to 100 mg/L. TFC (mean of three readings) was expressed in milligrams of quercetin equivalents per gram of honey (mg QE/100 g of honey) for each honey sample.

### 2.5 Antioxidant properties

#### 2.5.1 DPPH free radical scavenging assay

Previously given method by Ferreira et al. (31) with some modifications (29) was followed to determine the scavenging properties of honey in presence of 2,2-diphenyl-1-picrylhydrazyl free radical. Briefly, 0.20 mM DPPH-methanol solution was prepared and different concentrations of water honey solutions were mixed with 2.7 mL of already prepared DPPH-methanol solution. After shaking vigorously, the mixture was kept for 60 min in dark and then absorptions measured at 517 nm by using a UV–Visible spectrophotometer (Systronics Double Beam Spectrophotometer: 2203) and ascorbic acid was used as standard. The radical-scavenging activity was measured as per cent inhibition or per cent DPPH discoloration following the formula:


$$\%\text{inhibition}=[\frac{\mathrm{Ab}(b)-\mathrm{Ab}(s)}{\mathrm{Ab}(b)}]\times 100$$

Where Ab(b) is absorbance of the blank and Ab(s) is absorbance of the sample value (mean of three replications) was expressed as the volumetric concentration of honey samples in μl/ml (v/v).

#### 2.5.2 ABTS^•+^ free radical scavenging assay

The assay was carried out according to the method given by Re et al. (32) with slight modifications. Briefly, the cation radical (2,2-azino-bis-3-ethylbenzthiazoline-6-sulphonic acid) was obtained by mixing 7 mM ABTS^•+^ solution with 2.45 mM potassium persulfate solution (1:1) and allowed to stand in the dark at room temperature for 16 h (33). Dilution of the already prepared ABTS^•+^ solution was done with the help of methanol to achieve approximately 0.7 absorbance at *λ* = 734 nm. Then 100 μL of honey solution at the graded concentrations was mixed with ABTS^•+^ (2 mL) in a cuvette and after 6 min its absorbance was recorded. Blank was prepared by taking same solvent instead of the sample and Trolox was used as standard. The per cent inhibition was measured by:


$$\%\text{inhibition}=[\frac{\mathrm{Ab}(b)-\mathrm{Ab}(s)}{\mathrm{Ab}(b)}]\times 100$$

Where Ab(b) is absorbance of the blank and Ab(s) is absorbance of the sample and value was expressed (mean of three replications) as the volumetric concentration of honey samples in μl/ml (v/v).

#### 2.5.3 Ferric reducing/antioxidant power (FRAP) assay

Antioxidants power of honey samples to reduce the ferric 2,4,6-tripyridyl-s-triazine complex [Fe(III)]3 + to ferrous com plex [Fe(II)]2 + in acidic medium by developing the blue-color was determined as standard method given by Benzie and Strain (34) with some modifications. FRAP reagent was prepared with 300 mM acetate buffer (pH 3.6, 25 mL), 10 mM TPTZ (2.5 mL) in 40 mM HCl, and 20 mM FeCl_3_.6H_2_O (2.5 mL) solution. The Trolox was used as standard and concentrations (10–50 mg/mL) were used to prepare the calibration curve (*R^2^* = 0.9912). Absorbance was recorded at *λ* = 593 nm in UV–vis spectrophotometer (Systronics Double Beam Spectrophotometer: 2203). Sample preparation was done following the steps given by Ibrahimi and Hajdari (28) where 2.5 g of each honey sample was mixed with 25 mL of distilled completely in water in a shaking water bath filtered through Whatman paper and stored at −18 °C for further analysis. An amount of 100 μL of such sample extract was then mixed with 100 μL of FRAP reagent and incubated at 37 °C for 1 h before taking the absorbance reading. The results were finally expressed (mean of three replications) as mg Trolox equivalent (TE)/ 100 g honey.

### 2.6 Evaluation of antimicrobial effect against human pathogenic bacteria

#### 2.6.1 Bacterial isolates

Pure cultures of six clinically relevant human pathogenic bacterial strains *viz*., *Vibrio cholerae* (MTCC 3906), *Shigella flexneri* (MTCC 1457), *Salmonella Typhi* (MTCC 3224), *Escherichia coli* (MTCC 9537) [gram negative], *Streptococcus mutans* (MTCC 890) and *Streptococcus pyogenes* (MTCC 1926) [gram positive] were obtained from the Microbial Type Culture Collection and Gene Bank (MTCC), Chandigarh, India for. All the bacterial strains were maintained in the Applied Microbiology Laboratory, Department of Forestry, North Eastern Regional Institute of Science and Technology (NERIST), Nirjuli, Arunachal Pradesh. Primary cultures were preserved on nutrient agar slants at 4 °C for short-term storage. For long-term preservation, 50% glycerol stocks of each bacterial strain were prepared and stored at −20 °C and −80 °C, following the protocol described by Hallman et al. (35).

#### 2.6.2 Preparation of media and bacterial cultures

Mueller-Hinton Agar (MHA) was prepared according to the manufacturer’s instructions and poured into sterile Petri dishes under aseptic conditions. Bacterial cultures were grown in Mueller-Hinton Broth (MHB) and adjusted to a turbidity equivalent to 0.5 McFarland standard (~1.5 × 10^8^ CFU/mL). A volume of 100 μL of each standardized bacterial suspension was evenly spread across the surface of the MHA plates using sterile cotton swabs. Plates were allowed to air dry briefly to ensure proper absorption.

#### 2.6.3 Agar well diffusion method

The antibacterial activity of honey extracts was assessed using the agar well diffusion method, following the procedures described by Allen et al. (36) and the Kirby-Bauer technique (37), with modifications based on Nigussie et al. (38). A circular 6 mm diameter well was punched on the plate aseptically with a sterile borer. The stock solutions of the each honey samples were prepared in DDW and introduced into the wells at concentrations of 250 μg/mL, 500 μg/mL, 750 μg/mL, and 1,000 μg/mL. Each well received 100 μL of the respective honey solution. DDW was taken as negative control and reference antibiotic (Streptomycin) as positive control and incubated for 12 h at 37 °C.

#### 2.6.4 Measurement of antibacterial activity

Post-incubation, antibacterial activity was determined by measuring the diameter of the zones of inhibition (ZOI) surrounding each well. Measurements were recorded in millimeters (mm) using a calibrated ruler, and all assays were performed in triplicate to ensure reproducibility.

### 2.7 Evaluation of cytotoxicity effect of honey against HeLa and HepG2 cancer cells

Among the four tested honey samples, the bee species honey that showed significant promising results in the parameters evaluated and mentioned above was further evaluated for its cytotoxicity effect against HeLa (human cervical carcinoma) and HepG_2_ (human hepatocellular carcinoma) cells lines.

#### 2.7.1 Cell culture

HeLa and HepG_2_ cells, obtained from American Type Culture Collection (ATCC, USA) were cultured in DMEM-F12 supplemented with 20% FBS, 3 mM L-glutamine, and antibiotics (500 U/mL penicillin, 500 μg/mL streptomycin, 1.25 μg/mL amphotericin-B) in a humidified incubator at 37 °C with 5% CO_2_. HepG_2_ cells were initially expanded in 25 cm^2^ flasks and then passaged using 0.25% trypsin–EDTA into 75 cm^2^ flasks at 2.3 × 10^4^ cells/cm^2^ upon reaching 80% confluence. The medium was changed after every 2 days. Experiments commenced 24 h post-seeding to prevent differentiation. A similar procedure was followed for HeLa cells, with potential adjustments for seeding density and flask size.

#### 2.7.2 Preparation of sample solutions

A 30% (w/v) honey stock solution was prepared by dissolving 3 g of honey in 10 mL of sterile distilled water. This stock solution was then serially diluted using Dulbecco’s Modified Eagle’s Medium (DMEM) (Gibco, Thermo Fisher Scientific, USA) to obtain the desired working concentrations of 1, 2.5, 5, 10, 15, 20, and 25% (w/v). For each dilution, the required volume of the stock solution was added to the appropriate volume of medium, ensuring thorough mixing. Finally, all honey solutions, including the undiluted 30% stock, were filter-sterilized using a 0.22 μm syringe filter to remove any potential contaminants before being used in the MTT assay.

#### 2.7.3 Cytotoxicity by MTT [3-(4,5-Dimethylthiazol-2-yl)-2,5-diphenyltetrazolium bromide] assay

Both HeLa and HepG_2_ cells were seeded at a density of 10^5^ cells per well in 100 μL of culture medium in clear bottom 96-well tissue culture plates. After 24 h of incubation to allow for cell attachment, the cells were treated with varying concentrations of honey (1, 2.5, 5, 10, 15, 20, 25, and 30% w/v) in triplicate. 20 μL of each honey solution was added to the respective wells. A control group without honey treatment was also included. The cells were then incubated for either 24 or 48 h. Following the treatment period, the medium was removed, and the cells were washed twice with PBS. 15 μL of MTT reagent (0.5 mg/mL in PBS) was added to each well, and the cells were incubated for 3 h at 37 °C. The MTT reagent was then removed, and 100 μL of DMSO was added to each well. The plates were gently mixed on an orbital shaker for 1 hour at room temperature to dissolve the formazan crystals. Finally, absorbance was measured at 570 nm using an absorbance plate reader to assess the cytotoxicity of the honey treatments on both cell lines at both time points.

#### 2.7.4 Morphological observation

HeLa and HepG_2_ cells were cultured in DMEM supplemented with 20% FBS, 3 mM L-glutamine, and antibiotics (500 U/mL penicillin, 500 μg/mL streptomycin, 1.25 μg/mL amphotericin-B) in a humidified incubator at 37 °C with 5% CO_2_. Upon reaching 80% confluence, both cell lines were passaged and seeded into appropriate culture vessels. After 24 h, the cells were treated with the IC_50_ concentrations of the honey samples determined from the MTT assay. A control group without honey treatment was included for each cell line (HeLa and HepG_2_) and timepoint (24 and 48 h). Morphological changes were observed at 24 and 48 h post-treatment using an Axiovert 200 M phase contrast microscope at 20x magnification. Images were captured using Axiovision Rel. 4.2 software. Morphological changes such as cell shrinkage, rounding, membrane blebbing, and detachment were noted and compared between treated and control groups to assess the effect of honey on cell morphology.

### 2.8 Statistical analyses

All physicochemical, elemental and antioxidant properties of honey samples were analyzed using a one-way analysis of variance (ANOVA) under a Completely Randomized Design (CRD), considering honey type as the single factor by using IBM SPSS statistical 21 (39). *Post hoc* analyses were done by using Duncan’s Multiple Range Test (DMRT) to compute the significant differences among means at *p* ≤ 0.05. PCA biplot and heatmap were generated by using ggplot2 library of RStudio 2024.09. Half maximal effective concentrations (EC_50_) were determined by fitting dose–response curves in OriginPro 8.5 software using four-parameter logistic model indicated in following equation (40). Model fitting was performed using the mean inhibition percentages calculated from three replicates at each concentration.


$$y=A_{1}+\frac{A_{2^{-}}A_{1}}{1+10^{(\text{logx}0-x)p}}$$

where, *A_1_* = Baseline, *A_2_* = Maximum response, *p* = slope, *x*^0^ = concentration at the inflection point (EC_50_ value) and y = response (% inhibition).

For antimicrobial assay tow way ANOVA were performed following CDR design and the interaction C. D. (*p* ≤ 0.05) were considered to compute significance difference among different types of honey at different concentrations by using online statistical software OPSTAT developed in Haryana Agricultural University, Hisar, India (link: http://opstat.somee.com/opstat/twofactor/twofactor.html). DMRT was performed to evaluate the significance level. In cytotoxicity assay, the viable cells of selected cell lines were performed in triplicate (*n* = 3), and the data were represented as the means ± standard error and dose–response curves were computed.

## 3 Results and discussion

### 3.1 Physiochemical and biochemical properties of honey

The physico-chemical parameters of the four honey types collected from the AAU apiary demonstrated variation, with many of the attributes showing statistically significant differences (*p* ≤ 0.05) (Table 1). The specific gravity of honey at 27 °C ranged narrowly between 1.38 ± 0.004 (*A. cerana*) and 1.411 ± 0.02 (*T. iridipennis*), indicating comparable density among samples, consistent with values reported for fresh, mature honey. Moisture content which is a critical parameter of quality honey, was found to be highest in *T. iridipennis* (22.75 ± 0.24%) significantly, followed by *A. cerana* (20.92 ± 0.39%) and *A. mellifera* (20.68 ± 0.39%), while *A. dorsata* exhibited the lowest moisture level (19.00 ± 0.25%). The total reducing sugar content varied significantly where *A. dorsata* honey registered the highest value (74.07 ± 1.23%), followed by *T. iridipennis* (71.05 ± 0.55%), *A. mellifera* (65.30 ± 0.71%) and *A. cerana* (64.75 ± 0.81%). Sucrose content, which serves as an indicator of honey ripeness, was highest in case of *A. cerana* honey (10.84 ± 0.20%) and *A. mellifera* (9.578 ± 0.07%), while *T. iridipennis* contained only 2.03 ± 0.04%, and *A. dorsata* showed no detectable sucrose. The fructose-to-glucose (F/G) ratio, influencing crystallization tendency, ranged from 0.85 ± 0.02 (*A. mellifera*) to 1.24 ± 0.65 (*T. iridipennis*), suggesting a lower crystallization rate for *T. iridipennis*. Total ash content was highest in *T. iridipennis* honey (0.30 ± 0.02%), moderate in *A. cerana* (0.22 ± 0.01%), and below detectable levels in *A. mellifera* and *A. dorsata*. Free acidity, linked to organic acid content, was highest in *A. cerana* (41.30 ± 0.49 mEq acid/100 g) and lowest in *T. iridipennis* (20.81 ± 0.52 mEq acid/100 g), all within permissible international limits. Hydroxymethylfurfural (HMF), an indicator of overheating or prolonged storage, remained far below the Codex limit (40 mg/kg) in all samples, with *A. dorsata* recording the highest level (2.04 ± 0.02 mg/kg) and *A. cerana* and *A. mellifera* honey at no detectable level.

**Table 1:** Physicochemical parameters of four types of honey collected from the apiary of AAU, Jorhat, Assam.

Sl. no	Parameters	Honey			
		*T. iridipennis*	*A. cerana*	*A. mellifera*	*A. dorsata*
1.	Specific gravity at 27 °C	1.411 ± 0.02^a^	1.380 ± 0.004^a^	1.390 ± 0.01^a^	1.40 ± 0.01^a^
2.	Moisture (%)	22.75 ± 0.24^a^	20.92 ± 0.39^b^	20.68 ± 0.39^b^	19.00 ± 0.25^c^
3.	Total reducing sugars (%)	71.05 ± 0.55^b^	64.75 ± 0.81^c^	65.30 ± 0.71^c^	74.07 ± 1.23^a^
4.	Sucrose (%)	2.03 ± 0.04^c^	10.84 ± 0.20^a^	9.578 ± 0.07^b^	BDL
5.	F/G Ratio	1.24 ± 0.65^a^	1.037 ± 0.01^b^	0.850 ± 0.02^c^	0.970 ± 0.01^b^
6.	Total Ash (%)	0.30 ± 0.02^a^	0.22 ± 0.01^b^	BDL	BDL
7.	Free acidity (mEq Acid/1000 g)	20.81 ± 0.52^d^	41.30 ± 0.49^a^	26.28 ± 0.53^b^	23.99 ± 0.48^c^
8.	HMF (mg/kg)	0.28 ± 0.01^b^	BDL	BDL	2.04 ± 0.02^a^
9.	Diastase activity (DN)	19.63 ± 0.48^a^	7.610 ± 0.01^b^	8.260 ± 0.19^b^	1.05 ± 0.01^c^
10.	Pollen count (count/g)	8566.33 ± 33.47^d^	9901.67 ± 30.90^c^	17584.33 ± 11.78^a^	11806.33 ± 46.19^b^
11.	Proline (mg/kg)	1286.05 ± 2.24^a^	192.20 ± 1.70^c^	335.32 ± 1.74^b^	172.22 ± 2.41^d^
12.	EC (mS/cm)	0.53 ± 0.01^b^	0.596 ± 0.03^a^	0.180 ± 0.01^d^	0.370 ± 0.01^c^
13.	pH	3.11 ± 0.05^b^	4.50 ± 0.08^a^	4.38 ± 0.11^a^	4.49 ± 0.01^a^
14.	Color	Dark amber	Dark brown	Light brown	Brown

Values in the same row having same superscripts are not significantly different (*p* ≤ 0.05), values are expressed as mean of three replicates ±S.E.

Diastase activity (DN), a marker of honey freshness and enzyme content, was highest in *T. iridipennis* (19.63 ± 0.48 DN) and lowest in *A. dorsata* (1.05 ± 0.01 DN), with *A. cerana* (7.61 ± 0.01 DN) and *A. mellifera* (8.26 ± 0.19 DN) showing moderate activity. Pollen count varied widely, from 8566.33 ± 33.47 count/g in *T. iridipennis* honey to 17584.33 ± 11.78 count/g in *A. mellifera* honey, along with *A. dorsata* honey that also showed a high pollen load (11806.33 ± 46.19). Proline content, a key biochemical indicator of honey maturity and quality that was exceptionally high in *T. iridipennis* honey (1286.05 ± 2.24 mg/kg), exceeding all other species by a wide margin. Electrical conductivity was highest in *A. cerana* honey (0.596 ± 0.03 mS/cm) and lowest in *A. mellifera* honey (0.18 ± 0.01 mS/cm). pH values ranged between 3.11 ± 0.05 (*T. iridipennis*) and 4.50 ± 0.08 (*A. cerana*), confirming the acidic nature of honey which inhibits microbial growth. In terms of color classification, *T. iridipennis* honey was identified as dark amber, *A. cerana* as dark brown, *A. mellifera* as light brown, and *A. dorsata* as brown, reflecting differences in botanical origin and presence of different bioactive compounds.

**Figure 2:**
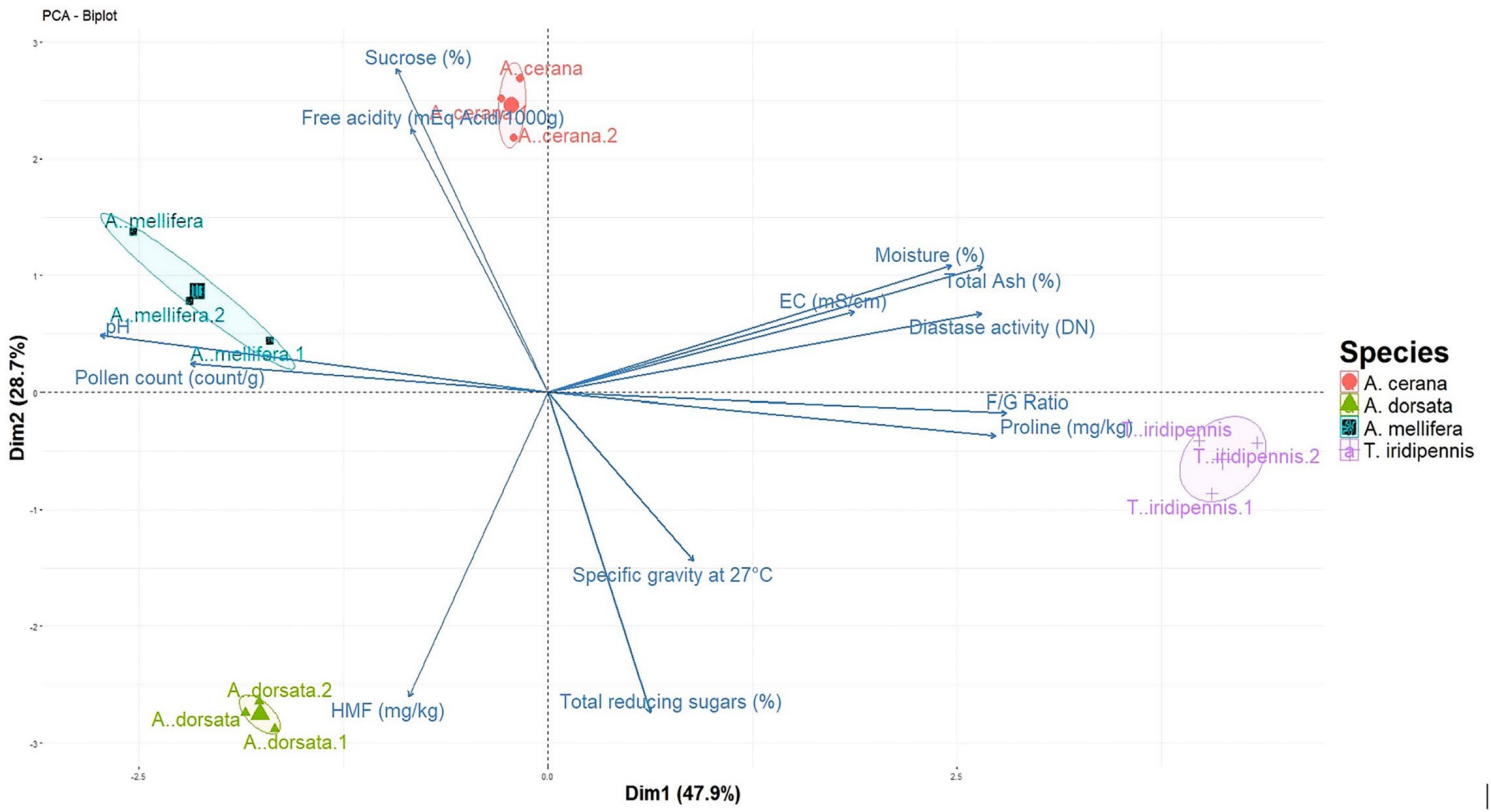
PCA biplot of physicochemical parameters of four types of honey collected from the apiary of AAU, Jorhat, Assam. Points show sample replicates for *Apis cerana*, *A. dorsata*, *A. mellifera*, and *Tetragonula iridipennis*; Longer vectors indicate better representation on the PC1-PC2 plane.

PCA of physico-chemical parameters showed that PC1 and PC2 explained 47.91 and 28.74% of total variance, respectively (76.65% cumulative) (Figure 2; Supplementary Table S1). PC1 loaded strongly and positively on F/G ratio (cos^2^ = 0.895), total ash (0.802), diastase activity (0.800) and moisture (0.692), and negatively on pollen count (−0.543). PC2 was driven mainly by sucrose (cos^2^ = 0.874) and free acidity (0.579) on the positive side, and by total reducing sugars (−0.854) and HMF (−0.771) on the negative side. The biplot separated honeys by species where *T. iridipennis* honey scored highly on PC1, associated with elevated moisture, ash, diastase activity, F/G ratio, and proline content. *A. cerana* honey was positioned along positive PC2 due to higher sucrose and free acidity content. Honey of *A. mellifera* plotted in the negative PC1/positive PC2 quadrant, linked with higher pH and pollen count. Further, *A. dorsata* honey was located in the negative PC1/negative PC2 quadrant, driven by higher HMF content and generally lower values for other parameters.

The physicochemical and biochemical characterization of honeys collected from four bee species under AAU apiary conditions at Jorhat revealed clear species-specific chemical fingerprints, strongly shaped by both bee physiology and floral foraging ecology. Stingless bees (*Tetragonula iridipennis*), due to their ability to exploit small flowers of herbs, weeds, and medicinal plants less frequently visited by *Apis* species (41), produced honey with very high moisture, ash, proline, and diastase activity, along with very low sucrose and an elevated fructose-to-glucose ratio. This pattern reflects complete enzymatic inversion of sucrose but incomplete dehydration- a hallmark of pot-stored honeys in humid conditions. The exceptionally high proline and diastase activity underscore strong bee-derived enzymatic and nitrogenous input, while the dark amber color and mineral richness are consistent with stingless bee honeys previously reported from Kerala (42), Tamil Nadu (43), and Karnataka (44). In contrast, *Apis dorsata* honey, derived largely from forest and wild flora, was the driest and most sugar-concentrated, with high reducing sugars and undetectable sucrose, reflecting maximally ripened nectar in open combs. However, diastase activity was extremely low despite negligible HMF, suggesting enzyme depletion arises from species-intrinsic physiology or environmental exposure in open nests (UV, temperature) rather than post-harvest heating. Similar enzyme-poor yet sugar-rich profiles have been documented in *A. dorsata* honeys from Maharashtra (45) and Karnataka (46).

*Apis cerana* honey, typically foraging on agricultural and horticultural crops, displayed elevated sucrose, electrical conductivity, and free acidity, combined with the highest pH of all four species. These traits suggest incomplete inversion of nectar sugars coupled with a strong organic acid-mineral buffering system. The dark-brown color matches a denser phenolic and mineral-ion profile. Such acidity and sucrose signatures align with reports from Himachal Pradesh (47) and South India (44). Importantly, Mahnot et al. (48) recorded Jorhat honey with 22.8% moisture, pH 3.37–4.00, free acidity of 25.39 meq/kg, and HMF up to 136.45 mg/kg. While our Jorhat samples partly overlapped with these ranges, they showed higher free acidity in *A. cerana* (41.3 meq/kg) and negligible HMF across all species, highlighting the advantages of controlled, species-verified apiary sampling compared to pooled surveys, and the influence of harvest stage and storage conditions. Further, *Apis mellifera* honey represented the lightest profile, with low conductivity, moderate moisture, lower reducing sugars, and higher sucrose relative to *A. dorsata*. This composition points to earlier harvest or slower inversion kinetics, as also noted for *A. mellifera* honeys in Assam, Meghalaya, and North Eastern Hill parts of this region (48, 49). The very high pollen counts in our Jorhat *A. mellifera* honeys reinforce its broad floral foraging, a feature also reported in Ethiopian honeys (50) and in *A. mellifera* versus stingless bee honeys from Trinidad and Tobago (11). The PCA substantiated these mechanistic groupings: PC1 captured an enzymatic-mineral-moisture axis dominated by stingless bee honey, while PC2 separated honeys based on sucrose and acidity versus sugar ripening. Accordingly, *T. iridipennis* clustered with high diastase, proline, ash, and F/G ratio; *A. dorsata* with concentrated sugars but low enzymes; *A. cerana* with sucrose and acidity; and *A. mellifera* with pollen and pH.

Comparison with earlier studies further situates these findings. Our moisture ranges matched values from Meghalaya honeys (51, 52) and Assam (48), while free acidity in *A. cerana* exceeded earlier Jorhat data, and HMF remained markedly lower- demonstrating better preservation under apiary conditions (see Supplementary Table S2 for detailed comparison). International parallels are also evident: Saudi honeys characterized by high reducing sugars (53), Ethiopian honeys by wide EC and pH variation (50, 54), and Trinidad stingless honeys by elevated moisture (11). Overall, the AAU Jorhat honeys show distinct biochemical “fingerprints” reflecting both bee species and foraging ecology: stingless bee honeys are enzyme- and amino acid-rich but more aqueous; *A. dorsata* honeys are fully ripened and sugar-rich but enzyme-poor; *A. cerana* honeys are acidic and mineral-laden; and *A. mellifera* honeys are lighter with broad pollen spectra. These insights reinforce species-level authentication, highlight Jorhat as a reference site for honey characterization in NE India, and provide a framework for quality assurance and product valorization.

### 3.2 Elemental profiles of honey

The mineral profiles of the four honey types collected from the AAU apiary exhibited pronounced variation (*p* ≤ 0.05) (Supplementary Table S3). Among the tested honey samples, *T. iridipennis* honey consistently contained the highest concentrations in case of most of the analyzed minerals. The results showed elevated levels of Fe (0.62 ± 0.01 mg/100 g), Ca (9.75 ± 0.03 mg/100 g), K (57.82 ± 1.47 mg/100 g), Mg (3.02 ± 0.02 mg/100 g), Zn (0.05 ± 0.01 mg/100 g) and Na (8.11 ± 0.13 mg/100 g), far surpassing those in other three types of honey samples. Out of the seven minerals, Potassium was found to be the most abundant mineral in all honey types, with *T. iridipennis* containing nearly fivefold higher levels than *A. cerana* (11.90 ± 0.17 mg/100 g) and *A. dorsata* (10.70 ± 0.11 mg/100 g). In contrast, *A. mellifera* honey exhibited the highest Mn content (0.09 mg/100 g), closely followed by *A. cerana* (0.07 ± 0.01 mg/100 g), while *A. dorsata* honey showed at par values with *T. iridipennis*. Among all the honey bee species, *A. dorsata* honey generally had the lowest mineral concentrations, particularly for Ca (1.27 ± 0.01 mg/100 g) and Mg (0.41 ± 0.01 mg/100 g). The predominance of K, along with appreciable amounts of Ca and Na in *T. iridipennis*, underscores its potential nutritional superiority among the honey types studied.

**Figure 3:**
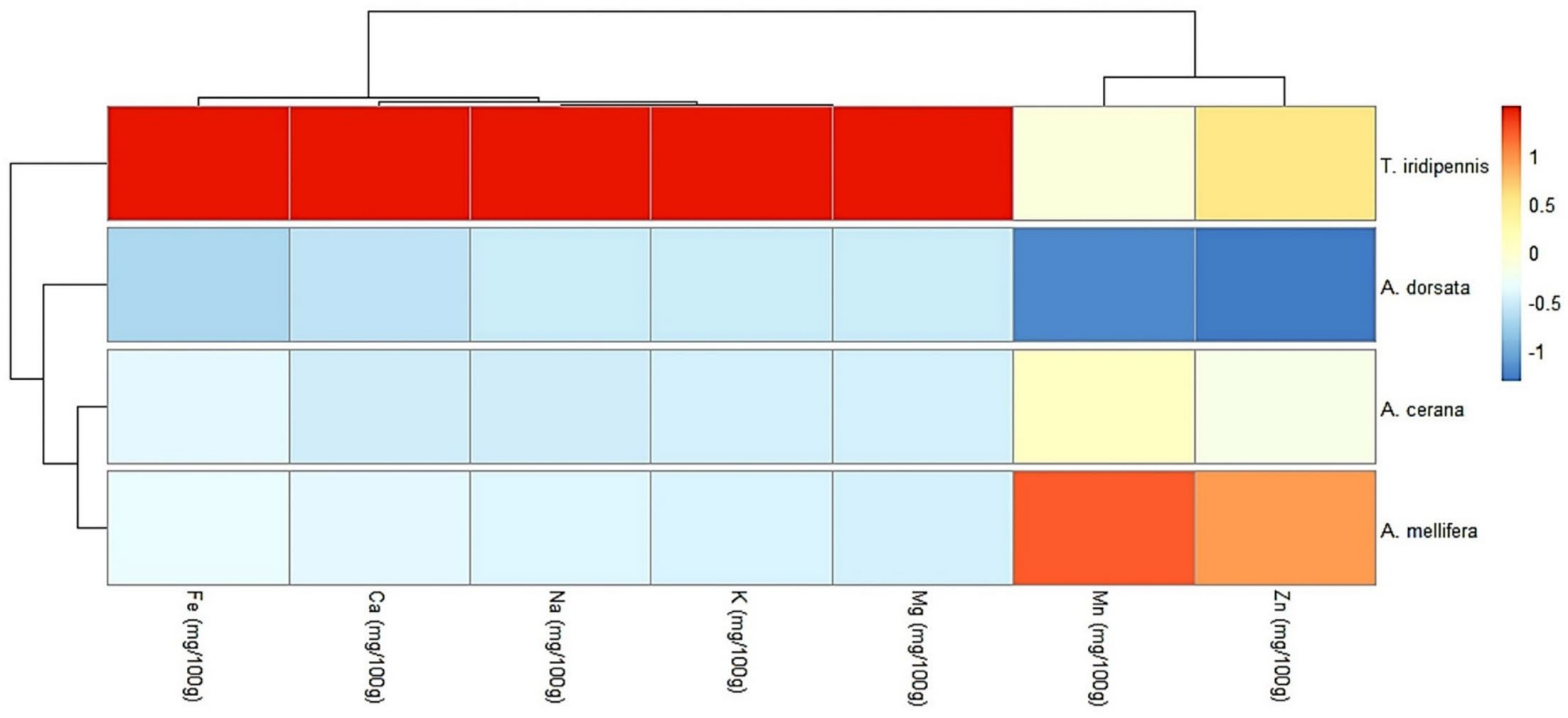
Heatmap showing hierarchical clustering of mineral contents in four types of honey collected from the apiary of AAU, Jorhat, Assam, dendrograms illustrate species and mineral groupings based on similarity in mineral profiles.

A hierarchical cluster heatmap of mineral content (Fe, Ca, Na, K, Mg, Mn, and Zn) revealed clear grouping patterns among the four honey bee species studied (Figure 3). Honey of *T. iridipennis* formed a distinct cluster characterized by consistently high concentrations of Fe, Ca, Na, K, and Mg (correlation score ≈ 1) compared to the other species. *A. dorsata* honey displayed moderately low levels of most minerals but was distinguished by notably low Zn and Mn content (correlation score ≈ − 1). Honey of *A. cerana* exhibited generally low mineral levels across all parameters, with slightly higher Mn compared to *A. dorsata*. Moreover, *A. mellifera* grouped separately due to its elevated Mn and Zn levels, despite showing low-to-moderate values for the remaining minerals. The dendrogram indicated two primary clusters: one comprising *T. iridipennis* (high Fe-K group) and the other containing the remaining three species, which further divided into an *A. mellifera* subgroup (high Mn-Zn profile) and a combined *A. dorsata* - *A. cerana* subgroup (low-mineral profile). These mineral composition differences likely reflect both species-specific foraging patterns and botanical sources of nectar.

In agreement with Mahnot (48), who reported potassium as the predominant mineral in honeys from Jorhat, our present study also recorded potassium as the most abundant element across all samples. Moreover, Nanda et al. (55) observed wide variation in the elemental profile of several types of North Indian honey of different botanical origin. Calcium content ranged from 33.7 mg/kg in commercial honey to 84.63 mg/kg in *Trifolium alexandrinum*, while sodium varied between 97.87 mg/kg in citrus flower honey and 304.30 mg/kg in mustard honey. Potassium was the most abundant element, ranging from 489.52 mg/kg in mustard honey to 932.56 mg/kg in multi-flower honey. Zinc levels varied from 2.55 mg/kg in citrus flower honey to 16.77 mg/kg in *T. alexandrinum*, while iron content ranged between 8.86 mg/kg (citrus honey) and 13.25 mg/kg (commercial honey). These results clearly demonstrate that honeys of different floral origin exhibit substantial heterogeneity in mineral content, much like the species-wise variation observed in the present study. For instance, the comparatively lower Ca and Mg values in *A. dorsata* honey from our results resemble the lower-end mineral profiles reported for certain commercial honeys by Nanda et al. (55), suggesting that floral preference and foraging range strongly govern mineral accumulation in honey. Moreover, Thakur et al. (47) further reported that the mineral composition of Himachal honey varied significantly across agro-climatic zones, with calcium ranging from 43.43 to 81.04 mg/kg, magnesium from 27.16 to 35.40 mg/kg, phosphorus from 43.57 to 62.93 mg/kg, potassium from 189.87 to 354.17 mg/kg, and sodium from 97.44 to 216.74 mg/kg. Potassium remained the most abundant mineral across all zones. This zonal variation mirrors the species-specific divergence observed in the present study, where *T. iridipennis* honey exhibited much higher Fe, Ca, K, and Mg compared to *Apis* honeys. Collectively, these findings underline that mineral levels are governed not only by bee species but also by the diversity of nectar sources and environmental conditions of the foraging landscape.

In southern India, Krishnappa and Sekarappa (46) reported exceptionally high mineral concentrations in stingless bee honey, with potassium (677.76–2298.30 mg/100 g) greatly exceeding all other minerals. While the present study also demonstrated that *T. iridipennis* honey is considerably more mineral-rich compared to *Apis* honeys, the absolute concentrations were lower than those reported from Karnataka. This deviation likely reflects differences in vegetation composition, nectar chemistry, and the specific foraging niches available to stingless bees across regions.

A comparison of raw and processed honeys from Kerala by Krishnasree and Mary Ukkuru (56) also confirmed interspecific variation, though their mineral distribution patterns showed some deviations from our present findings. They reported calcium ranging from 2.3 mg/100 g in *A. mellifera* to 4.22 mg/100 g in *A. dorsata*, sodium from 1.58 mg/100 g in *A. florea* to 5.67 mg/100 g in *A. mellifera*, and phosphorus from 2.20 mg/100 g in *A. florea* to 5.75 mg/100 g in *A. mellifera*. Potassium was consistently the most abundant mineral (30.50–52.00 mg/100 g), with *T. iridipennis* registering comparably high levels (47.50 mg/100 g). Interestingly, in their study *A. cerana* honey showed the highest iron content (1.42 mg/100 g), whereas in our results *T. iridipennis* was distinguished by higher Fe, Ca, and K. Similarly, zinc and manganese values reported from Kerala displayed wider ranges than those observed in the present study, and *T. iridipennis* exhibited the lowest iron levels (0.54 mg/100 g), which contrasts with our findings of its Fe-rich profile. Such deviations may be attributed to differences in regional floral sources, nectar chemistry, and foraging landscapes, which strongly influence mineral uptake. Nevertheless, both studies consistently highlight potassium as the dominant mineral across bee species, reaffirming its universal predominance in honey. On a broader scale, Mwangi et al. (57) reported similar findings in Kenyan stingless bee honey, where calcium, magnesium, sodium, and iron were present in comparatively small amounts, but potassium (17.94 ± 1.7 mg/L) remained the predominant mineral. Although the absolute concentrations differ due to geographic and unit scale variations, the trend of potassium predominance aligns closely with the present results on *T. iridipennis*, further supporting the conclusion that stingless bees consistently produce potassium-rich honey irrespective of geography.

Soil type and local geochemistry may substantially influence the mineral composition of nectar, and thereby the mineral profile of honey (48). Taken together, these studies highlight that mineral concentrations in honey are shaped by a complex interplay of bee species, floral origin, and agro-climatic factors. The consistent finding across diverse geographies is the predominance of potassium, a pattern corroborated in the present study as well as in earlier reports (46, 47, 55–57). The elevated potassium, calcium, and sodium levels recorded in *T. iridipennis* honey underscore its nutritional distinctiveness and reinforce the role of floral sources and stingless bee foraging behavior in shaping superior mineral profiles. Such findings also align with the broader conclusion of Saeed and Jayashankar (96), who emphasized potassium as the predominant mineral in Indian honey.

### 3.3 TPC, TFC and antioxidant properties of honey

**Figure 4:**
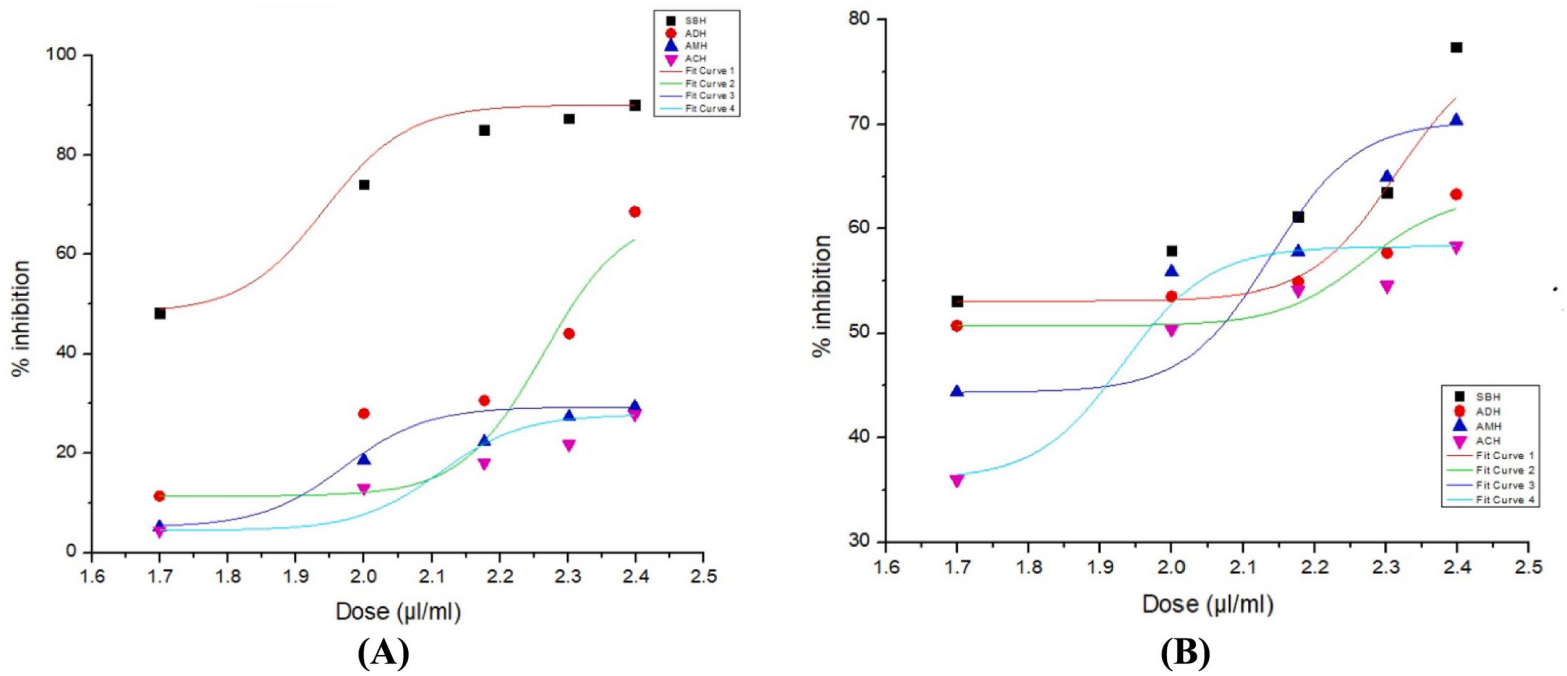
Dose–response curves for honey samples: **(A)** DPPH and **(B)** ABTS assays SHB, Stingless bee honey; ADH, *Apis dorsata* honey; AMH, *A. mellifera* honey, ACH, *A. cerana* honey.

The total phenolic content (TPC) of each of the tested honey samples varied significantly (*p* ≤ 0.05), ranging from 12.23 ± 0.03 mg GAE/100 g in *A. cerana* honey to 84.24 ± 0.58 mg GAE/100 g in *T. iridipennis*, which recorded the highest TPC. Similarly, total flavonoid content (TFC) was found to be highest in *T. iridipennis* (21.20 ± 0.51 mg QE/100 g) and lowest in case of *A. cerana* (6.49 ± 0.15 mg QE/100 g). The ferric reducing antioxidant power (FRAP) assay also indicated maximum antioxidant capacity in stingless bee honey (56.39 ± 0.21 mg TE/100 g), followed by *A. mellifera* (19.29 ± 0.02 mg TE/100 g), while *A. cerana* exhibited the lowest value (10.75 ± 0.01 mg TE/100 g) (Table 2). In the DPPH assay, the lowest EC_50_ value, indicating the strongest radical scavenging activity, was observed for *T. iridipennis* honey (51.55 μL/mL), whereas *A. cerana* exhibited the weakest activity (582.34 μL/mL). A similar trend was evident in the ABTS assay, where *T. iridipennis* honey again showed the lowest EC_50_ (47.23 μL/mL), while *A. cerana* recorded the highest (123.69 μL/mL). Honey from *A. dorsata* ranked second in both assays (EC_50_: 198.96 and 53.66 μL/mL for DPPH and ABTS, respectively), followed by *A. mellifera* (EC_50_: 568.18 and 73.56 μL/mL, respectively) (Table 2; Figure 4). Overall, *T. iridipennis* honey demonstrated superior phenolic content, flavonoid content, and antioxidant activity compared to the other three honey types.

**Table 2:** Total phenol, flavonoid and antioxidant properties of four types of honey collected from the apiary of AAU, Jorhat, Assam.

Parameters	*T. iridipennis*	*A. cerana*	*A. mellifera*	*A. dorsata*
TPC (mg GAE/100 g)	84.24 ± 0.58^a^	12.23 ± 0.03^d^	41.43 ± 1.02^c^	68.28 ± 0.98^b^
TFC (mg QE/100 g)	21.20 ± 0.51^a^	6.49 ± 0.15^c^	7.16 ± 0.11^c^	9.04 ± 0.16^b^
FRAP (mg TE/ 100 g)	56.39 ± 0.21^a^	10.75 ± 0.01^d^	19.29 ± 0.02^b^	13.23 ± 0.29^c^
DPPH [EC_50_ (μl/ml)]	51.55	582.34	568.18	198.96
ABTS [EC_50_ (μl/ml)]	47.23	123.69	73.56	53.66

Values in the same row having same superscripts are not significantly different (*p* ≤ 0.05), values are expressed as mean of three replicates ±S.E., EC₅₀ values (DPPH, ABTS) are reported as point estimates only. Lower EC₅₀ values denote higher antioxidant activity.

The present study demonstrated that stingless bee honey, particularly *T. iridipennis* collected from Assam, contained significantly higher TPC, TFC, and antioxidant capacity compared to honeys derived from *Apis* species. These results are consistent with earlier observations from other North Eastern states of India, where Nidhi et al. (49) reported marked interspecific differences in honey from the hill regions. In their study, *A. florea* exhibited the lowest TPC (677.60 mg GAEs/kg), while *A. mellifera* showed moderately higher levels (704.40 mg GAEs/kg). *A. cerana himalaya* and *A. dorsata* displayed comparable phenolic content (785.21 and 791.42 mg GAEs/kg), whereas stingless bee honeys (*Lepidotrigona arcifera* and *Tetragonula* sp.) recorded the highest phenolic concentrations (886.15 and 847.18 mg GAEs/kg). Moreover, stingless bee honeys in their study exhibited superior antioxidant activities in the DPPH assay.

By contrast, the present study provides the first species specific evidence from Assam, where *T. iridipennis* honey exhibited the highest phenolic (84.24 mg GAE/100 g) and flavonoid (21.20 mg QE/100 g) content along with the strongest antioxidant activities (lowest EC_50_ values in both DPPH and ABTS assays). This reinforces the broader pattern reported from other NE states, while adding state-specific data that highlights Assam as a potential hotspot for high-antioxidant stingless bee honey. Importantly, all four honey types were collected during the same flowering season, thereby minimizing seasonal bias. The observed variation among species is therefore more likely attributable to differences in floral resource utilization, bee physiology, and storage mechanisms. Stingless bees such as *T. iridipennis* are known to store honey in cerumen pots composed of beeswax and plant resins, which may leach additional phenolic and flavonoid compounds into the honey, thereby enhancing its antioxidant profile. In addition, stingless bees, owing to their smaller body size, frequently forage on floral resources that are less accessible to *Apis* species, including the small blossoms of herbs, weeds, and medicinal plants, which are often rich in bioactive phytochemicals (41). Notably, the stingless bee honey in this study was characterized as “dark amber” (Section 3.1), a color attribute that is commonly associated with higher phenolic content and stronger antioxidant capacity, thereby reinforcing the unique biochemical profile of this honey. These factors collectively explain why stingless bee honey demonstrates superior antioxidant potential compared to *Apis*-derived honeys, despite being collected from the same region and season. Although EC_50_ values reported in earlier studies are expressed in different units depending on the protocol used, the overall pattern of antioxidant activity remains comparable. These findings also align with the nationwide dataset reported by Saeed et al. Saeed et al. (29), who analyzed 64 Indian honey samples and observed wide variation in TPC (16.30–419.30 mg GAE/kg), TFC (15.70–240.90 mg QE/kg), and antioxidant activities, with IC_50_ values ranging from 6.30–130.40 mg/mL (DPPH) and 14.60–144.30 mg/mL (ABTS•+). The current results fall well within these ranges, further validating the robustness of antioxidant diversity in Indian honeys.

Comparative findings from international studies corroborate present patterns. Piljac-Žegarac et al. (58) found that monofloral honeys contained an average of 42.24 mg GAE/100 g phenolics with a FRAP value of 82.31 μM Fe(II), while heterofloral honeys exhibited higher phenolic content (58.75 mg GAE/100 g) and FRAP activity (157.66 μM Fe(II)), alongside enhanced radical scavenging properties. Similarly, Chua et al. (59) documented TPC values of 110.39–196.50 mg GAE/100 g and TFC values of 18.51–32.89 mg RE/100 g in three honeys of distinct botanical origin. Brazilian honey samples analyzed by Bueno-Costa et al. (60) demonstrated phenolic content between 11.37–54.01 mg GAE/100 g, flavonoids from 2.97–10.46 mg QE/100 g, ABTS activity from 8.24–111.48 mg trolox/100 g, and DPPH activity from 2.48–17.21 mg QEA/100 g. Likewise, Meda et al. (30) reported TPC values of 32.59–114.75 mg GAE/100 g and TFC values of 0.17–7.13 mg QE/100 g across 27 honey samples. Suriyatem et al. (33) recorded EC_50_ values of 276.70 mg/mL (DPPH) and 92.29 mg/mL (ABTS), highlighting variability across assays. In the Kosovo region, Ibrahimi and Hajdari (28) evaluated 100 honey samples and observed TPC ranging from 25.76–84.17 mg GAE/100 g, TFC from 1.11–7.51 mg CE/100 g, FRAP activity from 3.65–22.39 mg TE/100 g, and DPPH activity from 0.73–2.61 mg TE/100 g. Moreover, Stagos et al. (61) reported IC_50_ values of 7.5–109.0 mg/mL (DPPH) and 4.5–81.0 mg/mL (ABTS) in 21 honey samples from Mount Olympus, Greece, while Kaya and Yıldırım (62) recorded DPPH (EC_50_ values) of 85.25–98.29 μg/mL in phenolic extracts of five honey types. Overall, the results highlight stingless bee honey as a valuable functional food resource, with *T. iridipennis* from Assam showing distinct antioxidant superiority among the tested honeys.

### 3.4 Antimicrobial potency against some human pathogenic bacteria

**Figure 5:**
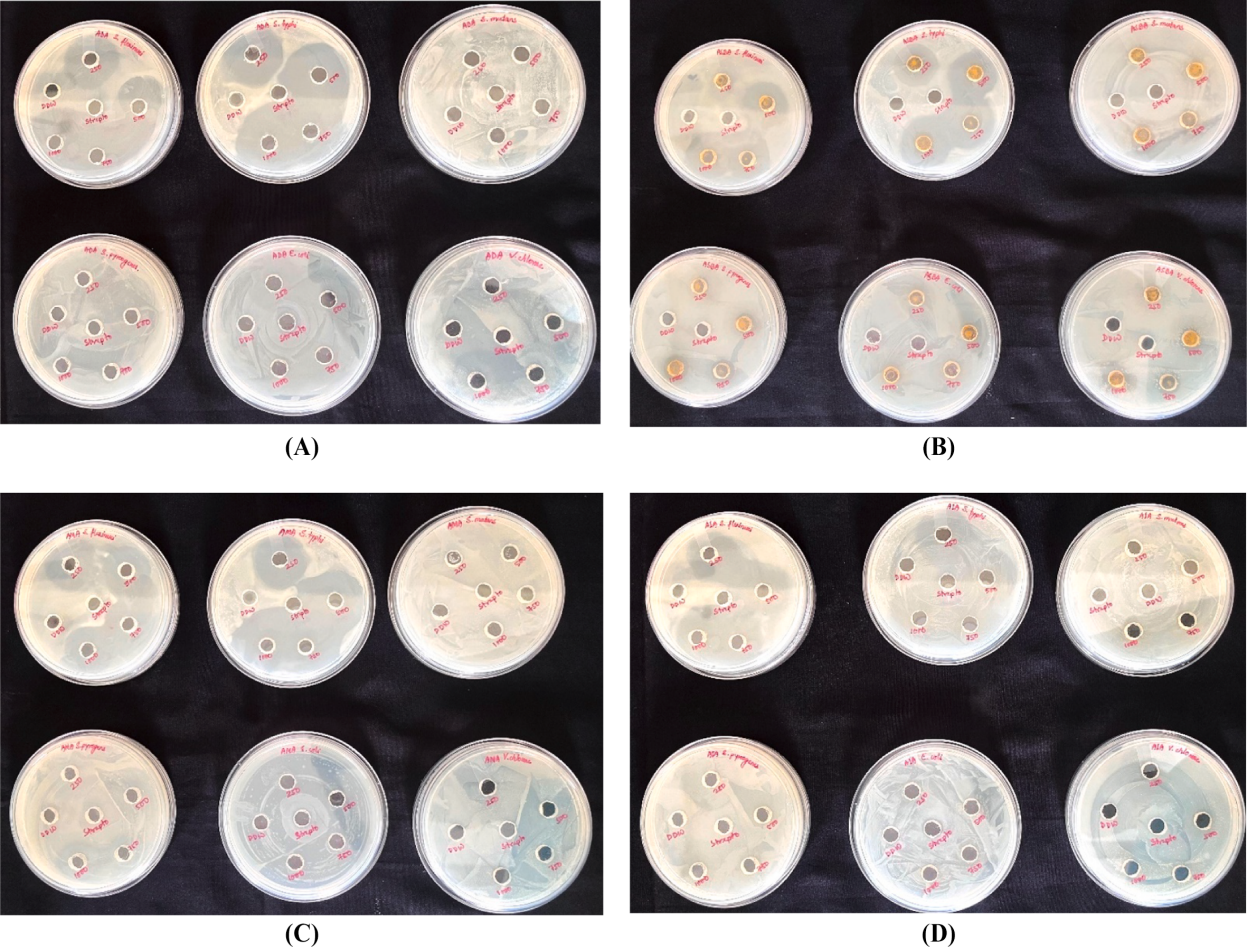
Zone of inhibition showing four types of honey against human-pathogenic bacteria in well diffusion assay **(A)** *A. dorsata,* **(B)** *T. iridipennis*, **(C)** *A. mellifera*, and **(D)** *A. cerana.*

**Figure 6:**
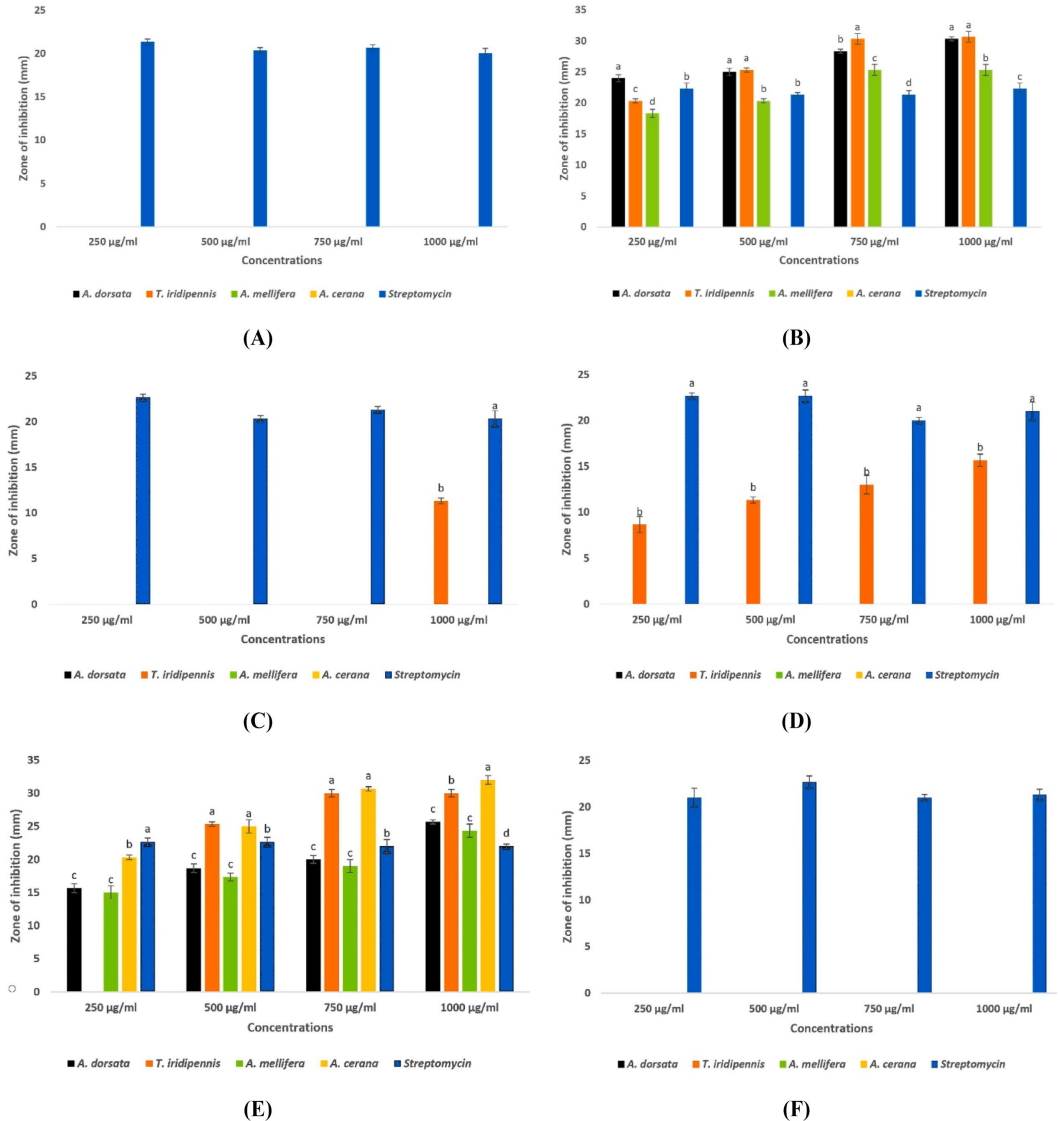
Zone of inhibition (mm) showing four types of honey against six human-pathogenic bacteria in well diffusion assay **(A)** *E. coli* **(B)** *S. typhi* **(C)** *S. mutans* **(D)** *S. pyrogenes* **(E)** *S. flexneri* **(F)** *V. cholerae*. Means of treatments within each concentration followed by the same lowercase letter are not significantly different at *p* ≤ 0.05 (DMRT concentration wise of each honey type); replication = 3.

Among the four honeys tested, *T. iridipennis* honey exhibited the broadest antimicrobial spectrum, inhibiting four pathogens: *Salmonella Typhi*, *Streptococcus pyogenes*, *Shigella flexneri*, and *Streptococcus mutans*. In contrast, *A. cerana* honey showed the narrowest activity, restricting inhibition to *S. flexneri* in the agar well-diffusion assay. Against *S. typhi*, honeys of *A. dorsata*, *T. iridipennis*, and *A. mellifera* produced significant zones of inhibition (ZOI), whereas *A. cerana* showed no activity. The highest ZOI was recorded for *T. iridipennis* (30.66 ± 0.88 mm), which was statistically at par with *A. dorsata* (30.33 ± 0.33 mm), while *A. mellifera* showed comparatively lower inhibition (25.33 ± 0.88 mm). All three were statistically comparable to the antibiotic control (22.33 mm) (CD = 1.67, *p* ≤ 0.05). Against *S. mutans*, inhibition was observed exclusively with *T. iridipennis* honey (11.33 ± 0.33 mm at 1000 μg/mL), which was statistically significant (CD = 0.71). A similar pattern was observed for *S. pyogenes*, where only *T. iridipennis* honey exhibited activity, with ZOI ranging from 8.67 ± 0.88 mm (250 μg/mL) to 15.67 ± 0.67 mm (1,000 μg/mL), all values exceeding the CD (1.27) (Supplementary Table S4; Figures 5, 6). In the case of *S. flexneri*, all four honeys were effective, though with variable potency. The strongest inhibition was produced by *A. cerana* (32.00 ± 0.67 mm at 1000 μg/mL), followed by *T. iridipennis* (30.00 ± 0.58 mm), *A. dorsata* (25.67 ± 0.33 mm), and *A. mellifera* (24.33 ± 1.00 mm). All observed values were significantly greater than the control (CD = 1.75). Notably, none of the honey samples inhibited *Escherichia coli* or *Vibrio cholerae* at the tested concentrations. Collectively, these findings highlight the superior antibacterial efficacy of *T. iridipennis* honey, which inhibited four clinically relevant pathogens, while *A. cerana* displayed the most limited spectrum, restricted to *S. flexneri* (Figures 5, 6).

The antibacterial spectrum of honey has been well documented against diverse human pathogens (10, 13, 57, 60–68). Consistent with these reports, our study demonstrated inhibition of *S. typhi*, aligning with earlier findings for both *S. typhi* and *S. typhi*murium (12, 60, 69, 70). Honey from Australian stingless bees has also shown notable antifungal and antibacterial effects, with hydrogen peroxide production persisting beyond 6 days in some samples (54).

However, the findings regarding *E. coli* remain inconsistent across studies. While several investigations reported strong inhibition (12, 13, 69, 70), others found minimal or no effect (57, 71, 72). Our results align with the latter, as none of the four honeys inhibited *E. coli*. Such divergence may reflect differences in honey type, floral source, physicochemical traits (pH, free acidity, sugar profile), peroxide/phenolic content, and the intrinsic tolerance of *E. coli* to honey’s stress factors. In agreement with prior reports, honey also inhibited *Shigella sonnei* and *S. flexneri* (70, 73, 74), and our results corroborate consistent activity against Gram-positive cocci such as *S. mutans* and *S. pyogenes* (75–81).

The antibacterial activity of honey arises from multiple complementary mechanisms. High sugar content generates osmotic pressure that dehydrates microbial cells (82), while its natural acidity (pH 3.2–4.5) creates an environment unfavorable for bacterial survival (82, 83). Upon dilution, glucose oxidase is activated, producing hydrogen peroxide at concentrations of 5–100 μg/g (≈0.146–2.93 mM), which correlates directly with antibacterial potency (10, 83–85). Diluted honey has also been reported to be more effective than undiluted honey against both Gram-positive and Gram-negative bacteria (67). In addition, phenolic compounds and flavonoids contribute to antimicrobial activity, with their associated antioxidant properties further enhancing efficacy (60, 62, 86). The extent of this activity largely depends on the botanical origin of nectar and prevailing environmental conditions (83, 87).

The superior antibacterial spectrum of *T. iridipennis* honey observed in this study can be explained by its distinctive physicochemical and biochemical attributes (Sections 3.1–3.3). Its low pH (3.11) and associated osmotic pressure restrict microbial growth by destabilizing cytoplasmic homeostasis and dehydrating bacterial cells. The exceptionally high proline content (1286.05 mg/kg) reflects honey maturity, while elevated diastase activity (19.63 DN) indicates freshness and overall enzymatic quality. Although not a direct marker of glucose oxidase, high diastase may suggest better preservation of enzymes required for hydrogen peroxide generation. The mineral composition of *T. iridipennis* honey, particularly its high potassium, calcium, and iron levels, may also contribute to antimicrobial activity by disrupting ionic balance, altering membrane permeability, and catalyzing redox reactions that intensify oxidative stress. Potassium and sodium ions can interfere with microbial enzyme systems, while divalent cations such as calcium and magnesium destabilize cell wall integrity.

Secondary metabolites are equally important. The markedly high phenolic (84.24 mg GAE/100 g) and flavonoid content (21.20 mg QE/100 g) in *T. iridipennis* honey, along with its superior antioxidant activity (FRAP, DPPH, and ABTS assays), strongly support the role of polyphenolic compounds in antimicrobial defense. These compounds may bind to bacterial membranes, increasing permeability and causing leakage of cellular contents. Flavonoids can intercalate with microbial DNA and inhibit nucleic acid synthesis, while phenolic acids may chelate essential metal ions required for bacterial metabolism. The strong antioxidant potential of this honey could also act synergistically with hydrogen peroxide, amplifying oxidative damage to microbial cells. By contrast, *A. cerana* honey, which showed the narrowest antimicrobial spectrum (restricted to *S. flexneri*), contained the lowest phenolic and flavonoid levels and exhibited weak antioxidant capacity. This highlights that, in addition to osmotic and acidic effects common to all honeys, the breadth and magnitude of antibacterial activity are strongly determined by phytochemical composition and mineral enrichment.

### 3.5 Evaluation of cytotoxicity effect of honey against HeLa and HepG2 cancer cells

As detailed in Sections [3.1–3.3], our results demonstrated that honey derived from *T. iridipennis* exhibited notable bioactive properties, particularly strong antioxidant activity. Therefore, among the four honey samples tested, *T. iridipennis* honey was selected for subsequent evaluation of its cytotoxic effects against HeLa and HepG_2_ cell lines.

**Figure 7:**
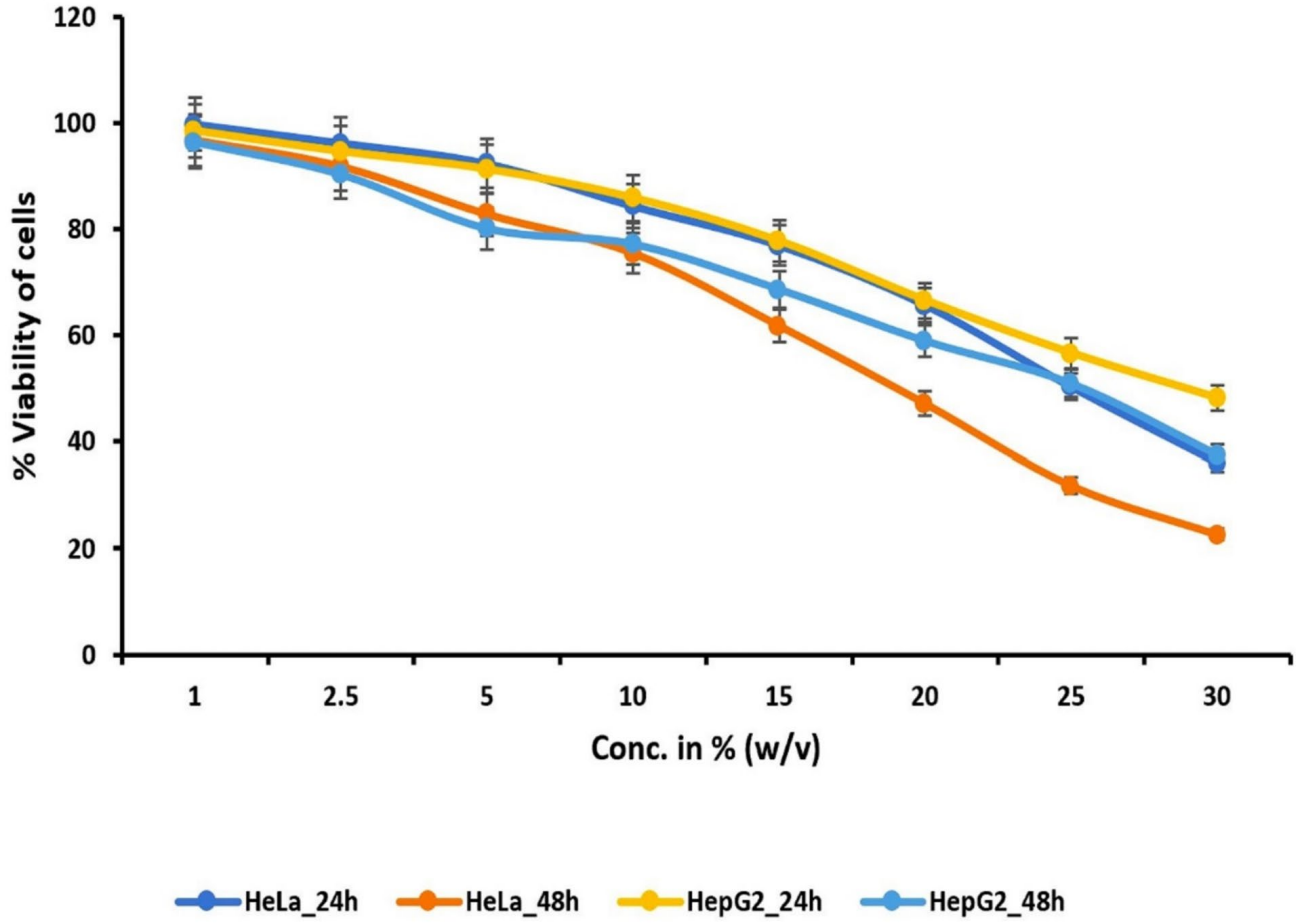
Dose–response curves on HeLa and HepG_2_ cell viability after 24 and 48 h of treatment with *T. iridipennis* honey (data points represent the mean ± SE (*n* = 3)).

**Figure 8:**
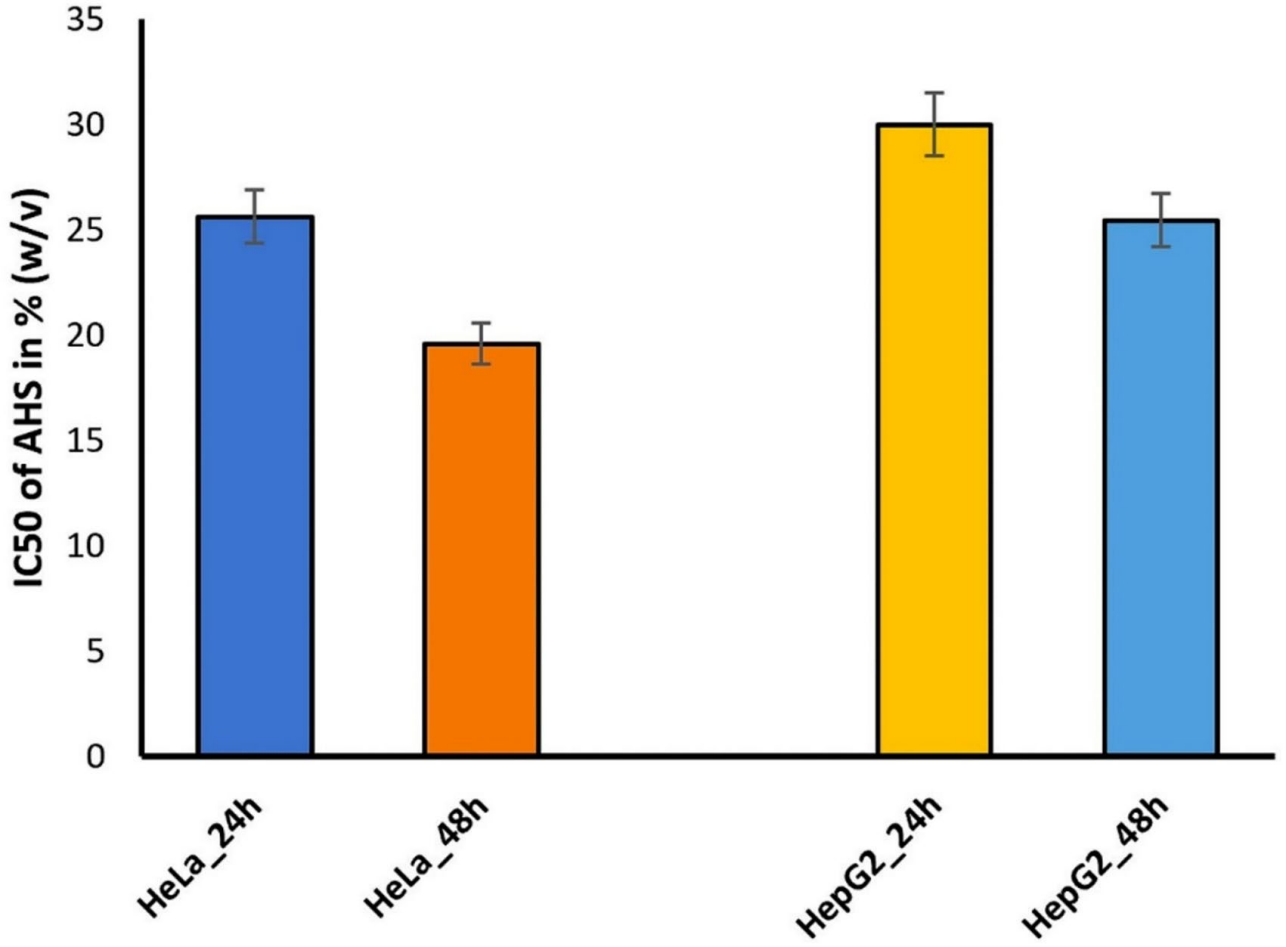
IC_50_ values of for HeLa and HepG_2_ cells after 24 and 48 h of treatment with *T. iridipennis* honey.

**Figure 9:**
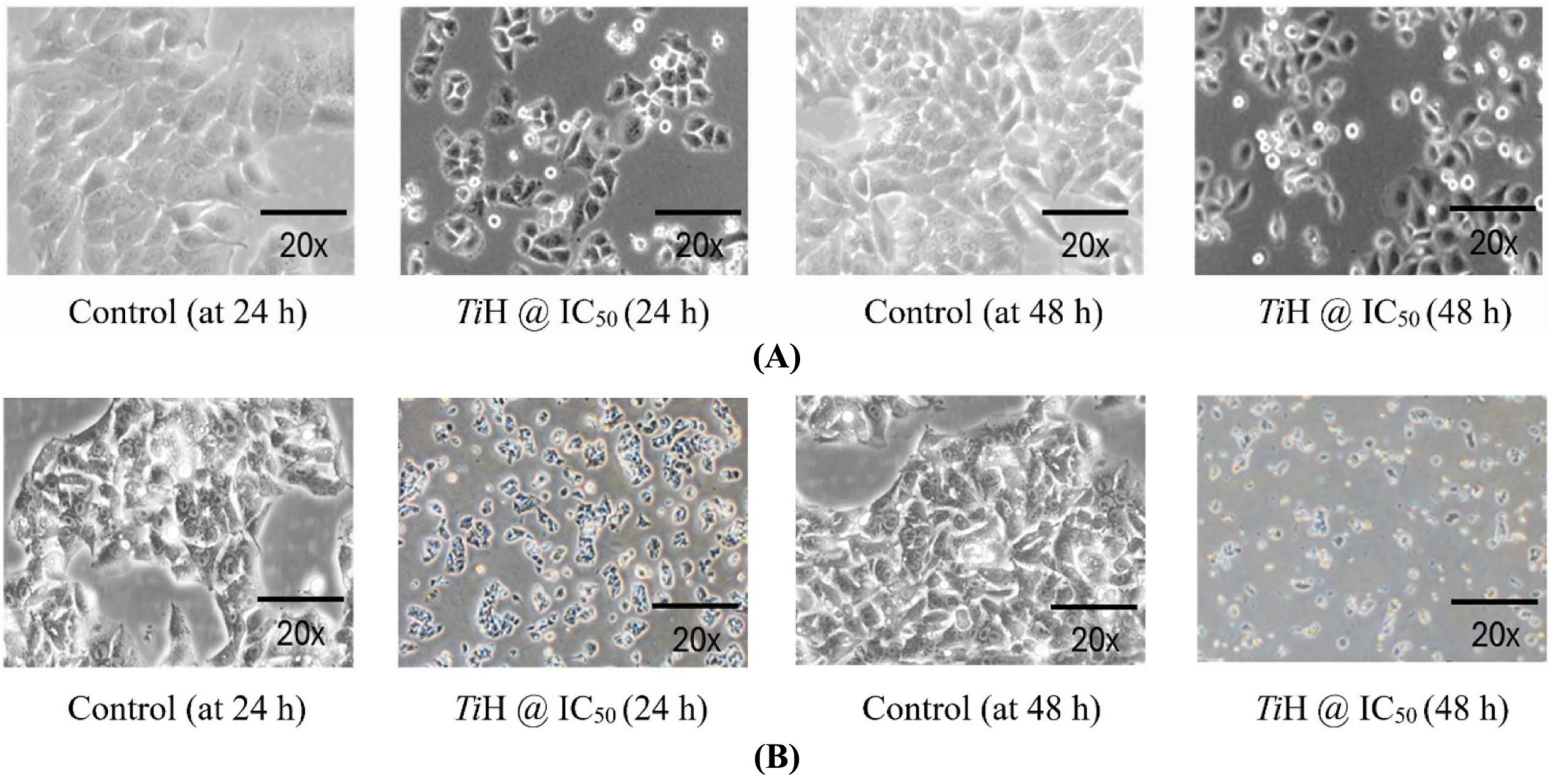
Morphological changes in **(A)** HeLa and **(B)** HepG_2_ cells treated with *T. iridipennis* honey (*Ti*H) at IC_50_ concentrations and control (without honey) for 24 and 48 h.

The MTT assay assessed the cytotoxic effects of *T. iridipennis* honey on HeLa and HepG_2_ cell lines after 24 and 48 h of treatment. The results indicate a dose-dependent and time-dependent decrease in cell viability for both cell lines (Figure 7; Supplementary Tables S5–S8). In HeLa cells, the IC_50_ values (concentration inhibiting 50% cell growth) were 25.62 ± 0.51% (w/v) at 24 h and 19.58 ± 1.85% (w/v) at 48 h (Figure 8). The results suggested that the cytotoxic effect of honey is more pronounced with longer exposure times. Similarly, in HepG_2_ cells, the IC_50_ values were 29.98 ± 0.21% (w/v) at 24 h and 25.45 ± 1.85% (w/v) at 48 h, indicating a time-dependent cytotoxicity increase (Figure 8). Morphological observation of HeLa and HepG_2_ cells treated with *T. iridipennis* at their respective IC_50_ concentrations revealed significant morphological alterations compared to untreated control cells. Both the cell lines clearly exhibited dose and time-dependent responses to the honey treatment, indicating its cytotoxic potential. These alterations included cell shrinkage, rounding, membrane blebbing, and detachment from the culture surface. These morphological changes were more pronounced after 48 h of treatment compared to 24 h, correlating with the results of the MTT assay (Figure 9). These observations further support the cytotoxic potential of *T. iridipennis* honey against both HeLa and HepG_2_ cancer cell lines. These findings suggested that the *T. iridipennis* honey possessed potential anticancer properties and need further investigation into its mechanisms of action and therapeutic applications.

The observed cytotoxicity is consistent with earlier studies reporting that honey exerts apoptotic and antiproliferative effects, induces cell cycle arrest, and modulates tumor necrosis factor (TNF). Honey has also been shown to act through caspase activation, regulation of p53, Bax and Bcl-2 proteins, and immune modulation (88). Mahmoud et al. (97) similarly demonstrated significant inhibition of HepG2 proliferation by 5–25% bee honey in a dose- and time-dependent manner, in agreement with the present study. Naik et al. (98) further showed that honey from Indian bees enhanced p53 expression and suppressed Cyclin B1 in HeLa cells, highlighting protein-level regulation as a possible mechanism of anticancer action. Screening by Kustiawan et al. (89) also confirmed the broad-spectrum cytotoxic potential of stingless bee honeys (*T. insica, T. apicalis, T. fuscobalteata,* and *T. fuscibasis*) across multiple human cancer cell lines, including colon, liver, gastric, lung, and breast cancers.

The therapeutic potential of bee honeys has been extensively reviewed. Rao et al. (90) highlighted the phenolics, flavonoids, vitamins, and enzymes responsible for antioxidant, anti-inflammatory, and anti-angiogenic actions, which contribute to their anticancer activity. Significant dose- and time- dependent cytotoxic effect of honey phenolic extracts in human prostate cancer cells (PC-3) was also reported by Kaya and Yıldırım (62). Moreover, studies with Malaysian Tualang honey (MTH) have shown induction of apoptosis via mitochondrial depolarization, caspase activation, angiogenesis inhibition, and cell cycle arrest in breast cancer models, while sparing normal cells. *In vivo*, MTH reduced tumor burden and improved histological and hematological parameters, and enhanced tamoxifen efficacy (88, 91).

Chemical profiling has identified unique compounds such as methyl syringate, fumaric acid, and 2-hydroxycinnamic acid associated with the anticancer activity of stingless bee honey (89, 92). Nahala et al. (92) demonstrated stronger radical scavenging and cytotoxic activity of *T. travancorica* honey compared to *A. indica* honey, with LCMS and molecular docking revealing selective binding to estrogen receptor *β*, suggesting a mechanism of action in reproductive cancers. Besides honey, other stingless bee products such as propolis and geopropolis also exhibit strong cytotoxicity. Arung et al. (93) reported that *H. fimbriata* propolis and honey showed higher activity than 5-fluorouracil, with mangiferonic acid identified as a key cytotoxic compound. Paz et al. (94) demonstrated potent cytotoxic and pro-apoptotic activity of geopropolis extracts from Brazilian *Melipona* species against hepatocellular carcinoma cell lines, with selectivity indices exceeding cisplatin. Nonetheless, certain studies have reported honeybee honey to be superior to stingless bee honey in terms of anticancer activity. Zulpa et al. (95) compared stingless bee honey and honeybee honey produced under identical environmental conditions in Malaysia. Honeybee honey exhibited higher phenolic content, stronger antioxidant capacity, and superior cytotoxicity against HeLa cells, suggesting that bee species and nectar source critically influence anticancer potential.

Importantly, the present results also provide scientific support for the reported ethnomedicinal use of stingless bee honey in Northeast India, where it is widely harvested from forestlands and traditionally employed as an ethnomedicine for treating burns, wounds, eye infections, diarrhea, and ulcers (18). Although these specific conditions were not directly evaluated in the present study, the demonstrated antioxidant, antimicrobial, and cytotoxic activities of *T. iridipennis* honey provide mechanistic support that may underlie such traditional applications, thereby strengthening the scientific rationale for its ethnomedicinal relevance, including its anticancer potential. The superior cytotoxic effects observed in HeLa and HepG2 cells can also be interpreted in light of the honey’s distinct physicochemical attributes such as high proline content and elevated diastase activity (Section 3.1), its mineral richness, particularly potassium, calcium, magnesium, and zinc (Section 3.2), and its exceptionally high phenolic and flavonoid contents along with strong radical scavenging activity (Section 3.3). These multifactorial features likely act synergistically to influence cellular redox balance and apoptotic pathways, thereby contributing to the cytotoxic responses observed. Taken together, the evidence demonstrates a clear continuum: bee species and floral source determine nectar composition; nectar composition governs physicochemical properties, mineral levels, and phenolic and flavonoid contents; these, in turn, drive antioxidant, antimicrobial, and cytotoxic activities; and ultimately, the richness of stingless bee honey translates into broad-spectrum therapeutic potential. While all honeys tested showed beneficial properties, *T. iridipennis* honey consistently outperformed the others, and to the best of our knowledge, this is the first systematic study from Northeast India to establish such scientific evidence, underscoring the need for further mechanistic and *in vivo* studies.

## 4 Conclusion

This study provides the first comprehensive, species-specific evaluation of the physicochemical attributes, antioxidant potential, and antimicrobial activity of honeys from *Apis cerana*, *A. mellifera*, *A. dorsata*, and *Tetragonula iridipennis* collected from Assam, Northeast India. Among the four species, *T. iridipennis* honey displayed a distinctly superior nutritional and bioactive profile, integrating high enzymatic activity, mineral richness, and abundant phenolic compounds. These compositional features were closely associated with enhanced antioxidant efficiency, broad-spectrum antibacterial activity, and notable *in-vitro* functional bioefficacy, demonstrating measurable effects on cell viability that support its potential health-promoting role. The findings scientifically substantiate traditional perceptions of stingless bee honey as a functional and health-promoting food while establishing a biochemical baseline for future research and product development. By linking traditional knowledge with modern analytical and cell-based validation, this work underscores the value of indigenous bee products in sustainable nutrition, biodiversity conservation, and functional food innovation. Furthermore, the study highlights the significance of apicultural and the growing emphasis on meliponiculture as models for environmentally sustainable and biodiversity-driven food production systems in this region. Advancing this line of research through molecular and applied investigations will further elucidate the mechanisms underlying honey bioactivity and promote its wider utilization in nutrition-oriented functional food development and sustainable apiculture practices.

## Data Availability

The original contributions presented in the study are included in the article/Supplementary material, further inquiries can be directed to the corresponding author.
