# Supplementary material for: Comparative nutritional and antioxidant profiling of Assam honeys: unveiling the untapped bioactivity of stingless bee honey
Source: Front Nutr. 2025 Dec 16;12:1737497. doi: 10.3389/fnut.2025.1737497 (PMC12751296; doi:10.3389/fnut.2025.1737497)
Supplement: Supplementary file 1 [file Table_1.DOCX]

**Supplementary Table S1. Summary statistics of PCA including eigenvalues, variance explained, and contributions of individuals and variables**

> summary(df_mat.pca)

Call:

PCA(X = df_mat, graph = TRUE)

Eigenvalues

Dim.1 Dim.2 Dim.3 Dim.4 Dim.5 Dim.6 Dim.7 Dim.8 Dim.9 Dim.10 Dim.11

Variance 6.228 3.737 2.103 0.722 0.104 0.063 0.024 0.016 0.003 0.001 0.000

% of var. 47.907 28.743 16.174 5.555 0.798 0.486 0.182 0.120 0.027 0.007 0.003

Cumulative % of var. 47.907 76.650 92.824 98.379 99.177 99.662 99.844 99.964 99.990 99.997 100.000

Individuals (the 10 first)

Dist Dim.1 ctr cos2 Dim.2 ctr cos2 Dim.3 ctr cos2

*T..iridipennis* | 4.154 | 3.981 21.208 0.919 | -0.409 0.372 0.010 | -0.459 0.834 0.012 |

*T..iridipennis*.1 | 4.397 | 4.060 22.053 0.853 | -0.865 1.667 0.039 | -0.770 2.350 0.031 |

*T..iridipennis*.2 | 4.436 | 4.336 25.151 0.955 | -0.428 0.409 0.009 | -0.418 0.692 0.009 |

*A..cerana* | 3.164 | -0.169 0.038 0.003 | 2.686 16.090 0.721 | 1.596 10.090 0.254 |

*A..cerana*.1 | 3.070 | -0.284 0.108 0.009 | 2.511 14.057 0.669 | 1.558 9.624 0.258 |

*A..cerana.*2 | 2.907 | -0.209 0.059 0.005 | 2.178 10.576 0.561 | 1.785 12.629 0.377 |

*A..mellifera* | 3.792 | -2.535 8.596 0.447 | 1.373 4.201 0.131 | -1.745 12.067 0.212 |

*A..mellifera.*1 | 3.185 | -1.700 3.865 0.285 | 0.440 0.432 0.019 | -2.400 22.830 0.568 |

*A..mellifera.*2 | 3.161 | -2.192 6.430 0.481 | 0.779 1.354 0.061 | -2.076 17.080 0.431 |

*A..dorsata* | 3.704 | -1.849 4.575 0.249 | -2.743 16.779 0.548 | 1.249 6.181 0.114 |

Variables (the 10 first)

Dim.1 ctr cos2 Dim.2 ctr cos2 Dim.3 ctr cos2

Specific gravity at 27°C | 0.300 1.448 0.090 | -0.486 6.328 0.236 | -0.208 2.048 0.043 |

Moisture (%) | 0.832 11.104 0.692 | 0.366 3.583 0.134 | -0.315 4.732 0.099 |

Total reducing sugars (%) | 0.211 0.714 0.044 | -0.924 22.846 0.854 | 0.193 1.771 0.037 |

Sucrose (%) | -0.312 1.559 0.097 | 0.935 23.379 0.874 | -0.114 0.623 0.013 |

F/G Ratio | 0.946 14.367 0.895 | -0.059 0.095 0.004 | 0.264 3.317 0.070 |

Total Ash (%) | 0.896 12.879 0.802 | 0.362 3.498 0.131 | 0.257 3.151 0.066 |

Free acidity (mEq Acid/1000g) | -0.282 1.278 0.080 | 0.761 15.485 0.579 | 0.561 14.947 0.314 |

HMF (mg/kg) | -0.287 1.321 0.082 | -0.878 20.644 0.771 | 0.371 6.542 0.138 |

Diastase activity (DN) | 0.894 12.843 0.800 | 0.228 1.386 0.052 | -0.372 6.568 0.138 |

Pollen count (count/g) | -0.737 8.716 0.543 | 0.081 0.177 0.007 | -0.667 21.170 0.445 |
