# Supplementary material for: Comparative nutritional and antioxidant profiling of Assam honeys: unveiling the untapped bioactivity of stingless bee honey
Source: Front Nutr. 2025 Dec 16;12:1737497. doi: 10.3389/fnut.2025.1737497 (PMC12751296; doi:10.3389/fnut.2025.1737497)
Supplement: Supplementary file 2 [file Table_2.DOCX]

**Supplementary Table S2. Comparative ranges of honey quality parameters as per the previous reports mentioned in the Results and Discussion section (3.1)**

**(Values are given as reported in respective studies)**

| **Parameter** | **Assam (including Jorhat) and Meghalaya**  **(Mahnot et al., 2018; Nidhi et al., 2025)**  **(NE India)** | **Meghalaya (Das, 2015; Kharkamni, 2021)**  **(NE India)** | **Himachal (Thakur, 2021)** | **Karnataka/Kerala (Divya 2018; Moulya 2023; Anusha 2020; Krishnappa 2024; Harithashree 2025)** | **Maharashtra (Sawarkar, 2025)** | **International (Sohaimy 2015; Kebebe 2019; Brown 2020)** |
| --- | --- | --- | --- | --- | --- | --- |
| Moisture (%) | 15.4 – 22.8 (Mahnot);  15.8-19.34 (Nidhi) | 16.6 – 19.4 (Kharkamni); 18.8 – 19.3 (Das) | 15.8 – 16.5 | stingless bee: 20.45 (Divya); 23.34 (Moulya); 15-30 (Anusha);  30.85-40.80 (Krishnappa)  *Apis*: 18.1-40.3 (Anusha); 24.5-23.6 (Harithashree) | 18.3 –19.5  (*A. cerana indica*); 19.1 – 21.3  (*A. dorsata*) | 14.7 – 18.3 (Sohaimy);  15.1 – 21.7 (Kebebe);  16.9 – 32.4 (Brown) |
| pH | 3.37 – 4.00 (Mahnot)  4.2 - 4.4 (Nidhi) | 3.59 – 3.73 (Kharkamni); 4.43 – 4.49 (Das) | 4.65 – 5.94 | stingless bee: 4.84 (Moulya); 3.7-5.9 (Anusha); 3.42-3.55 (Krishnappa);  *Apis*: 3.0-4.7 (Anusha); 3.1-3.5 (Harithashree) | 3.74 – 4.18  (*A. cerana indica*); 4.03 – 4.43  (*A. dorsata*) | 3.4 – 6.1 (Sohaimy); 3.5 – 4.5 (Kebebe); 2.88-3.17 (stingless bee) 3.91– 3.33 (*A. mellifera*) (Brown) |
| Electrical Conductivity (mS/cm) | 0.28 – 0.77 (Nidhi) | 0.51 – 0.61 (Kharkamni); 3.21 – 3.22 µS/cm (stingless bee: Das) | 0.11 – 0.24 | stingless bee: 1.32 dS/m (Moulya); 0.9-2.5 mS/cm (Anusha)  0.67-1.41 mS/cm (Krishnappa)  *Apis*: 0.16-1.08 mS/cm (Anusha) | 0.61 – 0.71  (*A. cerana indica*); 0.66 – 0.86  (*A. dorsata*) | 0.53 – 4.18 (Sohaimy);  0.45 – 1.55 (Kebebe) |
| Free Acidity (meq/kg) | 10.5 – 54.4 (Mahnot) | 32.3 – 42.0 (Kharkamni) | 32.8 – 46.1 | — | 30.0 – 33.8 (*A. cerana indica*); 32.4 – 36.8 (A. dorsata) | 10.5 – 54.4 (Sohaimy, global ranges) |
| Ash (% w/w) | 0.04 – 0.17 (Mahnot) | 0.003 – 0.005 (stingless: Das) | 0.04 – 0.17 | 0.172 (stingless bee, Moulya) | 0.22 – 0.36  (*A. cerana*)  0.37-0.43  (*A. dorsata*) | 0.18 – 0.80 (Kebebe)  0.23-2.33 (Sohaimy) |
| HMF (mg/kg) | 49.87 – 297.93 (Mahnot) | 8.9 – 34.2 (Kharkamni) | 55.9 – 70.2 | — | 22.1 – 28.34 (*A. cerana*)  23.37-31.61 (*A. dorsata*) | 0 – 40 (Codex); 297.9 in some NE honeys (Mahnot) |
| Diastase (DN) | — | — | 15.9 – 19.2 | 18 – 46 (stingless, Divya); 32.97- 58.5 (Harithasree, 2025) | 10.7 – 13.23 (*A. cerana*)  16.23-19.69 (*A. dorsata*) | — |
| Proline (mg/kg) | — | _ | — | 995 – 1513 (Divya, Kerala stingless) | 264 – 798 | — |
| Sugars | F/G: 0.81 – 1.54 (Mahnot) | — | F/G: 0.91 – 1.55 | Fructose 30–40; Glucose 11–17 (Krishnappa); sucrose 3–6 (*Apis cerana)* | Similar ranges as present | Saudi honey high reducing sugar (72, Sohaimy); F/G 0.42 - 2.35 (Sohaimy) |
