# Supplementary material for: Comparative nutritional and antioxidant profiling of Assam honeys: unveiling the untapped bioactivity of stingless bee honey
Source: Front Nutr. 2025 Dec 16;12:1737497. doi: 10.3389/fnut.2025.1737497 (PMC12751296; doi:10.3389/fnut.2025.1737497)
Supplement: Supplementary file 3 [file Table_3.DOCX]

**Supplementary Table S3. Comparative elemental analysis of *T. iridipennis*, *A. cerana*, *A. mellifera* and *A. dorsata* honey**

| **Sl. No** | **Elements** | **Honey (mg/100g)** | | | | **CD** |
| --- | --- | --- | --- | --- | --- | --- |
|  |  | ***T. iridipennis*** | ***A. cerana*** | ***A. mellifera*** | ***A. dorsata*** |  |
| 1. | Fe | 0.62±0.01^a^ | 0.14±0.01^b^ | 0.16±0.00^b^ | 0.07±0.00^c^ | 0.023 |
| 2. | Ca | 9.75±0.03^a^ | 1.60±0.01^c^ | 2.08±0.04^b^ | 1.27±0.01^d^ | 0.1 |
| 3. | Mn | 0.06±0.02^b^ | 0.07±0.01^ab^ | 0.09±0.00^a^ | 0.04±0.00^b^ | 0.02 |
| 4. | K | 57.82±1.47^a^ | 11.90±0.17^b^ | 12.20±0.03^b^ | 10.70±0.11^b^ | 2.46 |
| 5. | Mg | 3.02±0.02^a^ | 0.47±0.01^b^ | 0.48±0.01^b^ | 0.41±0.01^c^ | 0.05 |
| 6. | Zn | 0.05±0.01^a^ | 0.04±0.00^a^ | 0.05±0.00^a^ | 0.03±0.00^b^ | 0.01 |
| 7. | Na | 8.11±0.13^a^ | 0.93±0.02^c^ | 1.20±0.06^b^ | 0.87±0.01^c^ | 0.24 |
| Values in the same row having same superscripts are not significantly different (p<0.05), values are expressed as mean of three replicates ±S.E. | | | | | | |
