# Supplementary material for: Comparative nutritional and antioxidant profiling of Assam honeys: unveiling the untapped bioactivity of stingless bee honey
Source: Front Nutr. 2025 Dec 16;12:1737497. doi: 10.3389/fnut.2025.1737497 (PMC12751296; doi:10.3389/fnut.2025.1737497)
Supplement: Supplementary file 4 [file Table_4.DOCX]

**Supplementary Table S4. Well diffusion assay against six human-pathogenic bacteria with different concentrations of honey**

| **Bacteria** | **Treatment** | **ZOI (mm)** | | | | **CD (p=0.05)**  **Treatment × Concentration** |
| --- | --- | --- | --- | --- | --- | --- |
|  |  | **250** | **500** | **750** | **1000** |  |
| ***E. coli*** | *Apis dorsata* | 0 | 0 | 0 | 0 | - |
|  | *T. iridipennis* | 0 | 0 | 0 | 0 |  |
|  | *Apis mellifera* | 0 | 0 | 0 | 0 |  |
|  | *Apis cerana* | 0 | 0 | 0 | 0 |  |
|  | Streptomycin | 21.33 ± 0.33 | 20.33 ± 0.33 | 20.66± 0.33 | 20.00 ± 0.58 |  |
| ***S. typhi*** | *Apis dorsata* | 24.00 ± 0.58 | 25.00 ± 0.58 | 28.33 ± 0.33 | 30.33± 0.33 | 1.67 |
|  | *T. iridipennis* | 20.33 ± 0.33 | 25.33 ± 0.33 | 30.33 ± 0.88 | 30.66 ± 0.88 |  |
|  | *Apis mellifera* | 18.33 ± 0.67 | 20.33 ± 0.33 | 25.33 ± 0.88 | 25.33 ± 0.88 |  |
|  | *Apis cerana* | 0 | 0 | 0 | 0 |  |
|  | Streptomycin | 22.33 ± 0.88 | 21.33 ± 0.33 | 21.33 ± 0.66 | 22.33 ± 0.88 |  |
| ***S. mutans*** | *Apis dorsata* | 0 | 0 | 0 | 0 | 0.71 |
|  | *T. iridipennis* | 0 | 0 | 0 | 11.33 ± 0.33 |  |
|  | *Apis mellifera* | 0 | 0 | 0 | 0 |  |
|  | *Apis cerana* | 0 | 0 | 0 | 0 |  |
|  | Streptomycin | 22.67 ± 0.33 | 20.33 ± 0.33 | 21.33 ± 0.33 | 20.33 ± 0.88 |  |
| ***S. pyrogenes*** | *Apis dorsata* | 0 | 0 | 0 | 0 | 1.27 |
|  | *T. iridipennis* | 8.67 ± 0.88 | 11.33 ± 0.33 | 13.00 ± 1.00 | 15.67 ± 0.67 |  |
|  | *Apis mellifera* | 0 | 0 | 0 | 0 |  |
|  | *Apis cerana* | 0 | 0 | 0 | 0 |  |
|  | Streptomycin | 22.67 ± 0.33 | 22.67 ± 0.67 | 20.00 ± 0.33 | 21.00 ± 1.00 |  |
| ***S. flexneri*** | *Apis dorsata* | 15.67 ±0.67 | 18.67 ± 0.67 | 20.00 ± 0.58 | 25.67 ± 0.33 | 1.75 |
|  | *T. iridipennis* | 0 | 25.33 ± 0.33 | 30.00 ± 0.58 | 30.00 ± 0.58 |  |
|  | *Apis mellifera* | 15.00 ± 1.00 | 17.33 ± 0.58 | 19.00 ± 1.00 | 24.33 ± 1.00 |  |
|  | *Apis cerana* | 20.33 ± 0.33 | 25.00 ± 1.00 | 30.67 ± 0.33 | 32.00 ± 0.67 |  |
|  | Streptomycin | 22.67 ± 0.58 | 22.67 ± 0.67 | 22.00 ± 1.00 | 22.00 ± 0.33 |  |
| ***V. cholerae*** | *Apis dorsata* | 0 | 0 | 0 | 0 | - |
|  | *T. iridipennis* | 0 | 0 | 0 | 0 |  |
|  | *Apis mellifera* | 0 | 0 | 0 | 0 |  |
|  | *Apis cerana* | 0 | 0 | 0 | 0 |  |
|  | Streptomycin | 21.00 ± 1.00 | 22.67 ± 0.67 | 21.00 ± 0.33 | 21.33 ± 0.58 |  |
| Means of treatments within each concentration followed by the same lowercase letter are not significantly different at p ≤ 0.05 (DMRT concentration wise of each honey type); replication= 3 | | | | | | |
