# Supplementary material for: Comparative nutritional and antioxidant profiling of Assam honeys: unveiling the untapped bioactivity of stingless bee honey
Source: Front Nutr. 2025 Dec 16;12:1737497. doi: 10.3389/fnut.2025.1737497 (PMC12751296; doi:10.3389/fnut.2025.1737497)
Supplement: Supplementary file 5 [file Table_5.DOCX]

**Supplementary Table S5. Cytotoxicity of *T.* *iridipennis* honey against HeLa cells at 24 h treatment**

|  | **Absorbance @570 nm** | | | | **% Viability of HeLa cells at 24 h** | | | |
| --- | --- | --- | --- | --- | --- | --- | --- | --- |
| **Conc. in % (w/v)** | **Run-1** | **Run-2** | **Run-3** | **Mean absorbance** | **Run-1** | **Run-2** | **Run-3** | **Mean** |
| Control | 1.13 | 1.11 | 1.14 | 1.127 | 100 | 100 | 100 | 100 ± 0.00 |
| 1 | 1.12 | 1.13 | 1.12 | 1.123 | 99.12 | 101.80 | 98.25 | 99.72 ± 1.85 |
| 2.5 | 1.08 | 1.09 | 1.08 | 1.083 | 95.58 | 98.20 | 94.74 | 96.17 ± 1.80 |
| 5 | 1.03 | 1.04 | 1.05 | 1.040 | 91.15 | 93.69 | 92.11 | 92.32 ± 1.28 |
| 10 | 0.95 | 0.94 | 0.96 | 0.950 | 84.07 | 84.68 | 84.21 | 84.32 ± 0.32 |
| 15 | 0.87 | 0.85 | 0.88 | 0.867 | 76.99 | 76.58 | 77.19 | 76.92 ± 0.31 |
| 20 | 0.75 | 0.74 | 0.73 | 0.740 | 66.37 | 66.67 | 64.04 | 65.69 ± 1.44 |
| 25 | 0.58 | 0.57 | 0.55 | 0.567 | 51.33 | 51.35 | 48.25 | 50.31 ± 1.78 |
| 30 | 0.42 | 0.41 | 0.39 | 0.407 | 37.17 | 36.94 | 34.21 | 36.11 ± 1.64 |
| IC_50_ |  | | | | 26.20 | 25.40 | 25.25 | **25.62 ± 0.51** |

**Supplementary Table S6.** **Cytotoxicity of *T.* *iridipennis* honey against HeLa cells at 48 h treatment**

| **Conc. in % (w/v)** | **Absorbance @570 nm** | | | | **% Viability of HeLa cells at 24 h** | | | |
| --- | --- | --- | --- | --- | --- | --- | --- | --- |
|  | **Run-1** | **Run-2** | **Run-3** | **Mean absorbance** | **Run-1** | **Run-2** | **Run-3** | **Mean** |
| Control | 1.41 | 1.4 | 1.43 | 1.413 | 100 | 100 | 100 | 100 ± 0.00 |
| 1 | 1.38 | 1.35 | 1.37 | 1.367 | 97.87 | 96.43 | 95.80 | 96.70 ± 1.06 |
| 2.5 | 1.29 | 1.31 | 1.29 | 1.297 | 91.49 | 93.57 | 90.21 | 91.76 ± 1.69 |
| 5 | 1.17 | 1.15 | 1.19 | 1.170 | 82.98 | 82.14 | 83.22 | 82.78 ± 0.56 |
| 10 | 1.05 | 1.08 | 1.07 | 1.067 | 74.47 | 77.14 | 74.83 | 75.48 ± 1.45 |
| 15 | 0.89 | 0.88 | 0.85 | 0.873 | 63.12 | 62.86 | 59.44 | 61.81 ± 2.05 |
| 20 | 0.68 | 0.65 | 0.67 | 0.667 | 48.23 | 46.43 | 46.85 | 47.17 ± 0.94 |
| 25 | 0.46 | 0.45 | 0.44 | 0.450 | 32.62 | 32.14 | 30.77 | 31.85 ± 0.96 |
| 30 | 0.33 | 0.32 | 0.31 | 0.320 | 23.40 | 22.86 | 21.68 | 22.65 ± 0.88 |
| IC_50_ |  | | | | 19.60 | 19.76 | 19.37 | **19.58 ± 1.85** |

**Supplementary Table S7. Cytotoxicity of *T.* *iridipennis* honey against HepG2 cells at 24 h treatment**

|  | **Absorbance @570 nm** | | | | **% Viability of HepG2 cells at 24 h** | | | |
| --- | --- | --- | --- | --- | --- | --- | --- | --- |
| **Conc. in % (w/v)** | **Run-1** | **Run-2** | **Run-3** | **Mean absorbance** | **Run-1** | **Run-2** | **Run-3** | **Mean** |
| Control | 1.09 | 1.11 | 1.12 | 1.107 | 100 | 100 | 100 | 100 ± 0.00 |
| 1 | 1.08 | 1.09 | 1.1 | 1.090 | 99.08 | 98.20 | 98.21 | 98.50 ± 0.51 |
| 2.5 | 1.05 | 1.04 | 1.05 | 1.047 | 96.33 | 93.69 | 93.75 | 94.59 ± 1.51 |
| 5 | 1.01 | 1 | 1.02 | 1.010 | 92.66 | 90.09 | 91.07 | 91.27 ± 1.30 |
| 10 | 0.94 | 0.96 | 0.95 | 0.950 | 86.24 | 86.49 | 84.82 | 85.85 ± 0.90 |
| 15 | 0.88 | 0.86 | 0.84 | 0.860 | 80.73 | 77.48 | 75.00 | 77.74 ± 2.88 |
| 20 | 0.72 | 0.74 | 0.75 | 0.737 | 66.06 | 66.67 | 66.96 | 66.56 ± 0.46 |
| 25 | 0.63 | 0.61 | 0.64 | 0.627 | 57.80 | 54.95 | 57.14 | 56.63 ± 1.49 |
| 30 | 0.52 | 0.55 | 0.53 | 0.533 | 47.71 | 49.55 | 47.32 | 48.19 ± 1.19 |
| IC_50_ |  | | | | 29.94 | 30.20 | 29.79 | **29.98 ± 0.21** |

**Supplementary Table S8. Cytotoxicity of *T.* *iridipennis* honey against HepG2 cells at 48 h treatment**

|  | **Absorbance @570 nm** | | | | **% Viability of HepG2 cells at 48 h** | | | |
| --- | --- | --- | --- | --- | --- | --- | --- | --- |
| **Conc. in % (w/v)** | **Run-1** | **Run-2** | **Run-3** | **Mean absorbance** | **Run-1** | **Run-2** | **Run-3** | **Mean** |
| Control | 1.37 | 1.39 | 1.36 | 1.373 | 100 | 100 | 100 | 100 ± 0.00 |
| 1 | 1.32 | 1.34 | 1.31 | 1.323 | 96.35 | 96.40 | 96.32 | 96.36 ± 0.04 |
| 2.5 | 1.24 | 1.25 | 1.23 | 1.240 | 90.51 | 89.93 | 90.44 | 90.29 ± 0.32 |
| 5 | 1.11 | 1.09 | 1.1 | 1.100 | 81.02 | 78.42 | 80.88 | 80.11 ± 1.47 |
| 10 | 1.05 | 1.06 | 1.07 | 1.060 | 76.64 | 76.26 | 78.68 | 77.19 ± 1.30 |
| 15 | 0.94 | 0.95 | 0.94 | 0.943 | 68.61 | 68.35 | 69.12 | 68.69 ± 0.39 |
| 20 | 0.81 | 0.8 | 0.82 | 0.810 | 59.12 | 57.55 | 60.29 | 58.99 ± 1.37 |
| 25 | 0.7 | 0.69 | 0.71 | 0.700 | 51.09 | 49.64 | 52.21 | 50.98 ± 1.29 |
| 30 | 0.53 | 0.52 | 0.5 | 0.517 | 38.69 | 37.41 | 36.76 | 37.62 ± 0.98 |
| IC_50_ |  | | | | 25.67 | 24.95 | 25.73 | **25.45 ± 1.85** |
